# Genomic Survey of the Non-Cultivatable Opportunistic Human Pathogen, *Enterocytozoon bieneusi*

**DOI:** 10.1371/journal.ppat.1000261

**Published:** 2009-01-09

**Authors:** Donna E. Akiyoshi, Hilary G. Morrison, Shi Lei, Xiaochuan Feng, Quanshun Zhang, Nicolas Corradi, Harriet Mayanja, James K. Tumwine, Patrick J. Keeling, Louis M. Weiss, Saul Tzipori

**Affiliations:** 1 Division of Infectious Diseases, Department of Biomedical Sciences, Tufts Cummings School of Veterinary Medicine, North Grafton, Massachusetts, United States of America; 2 Marine Biological Laboratory, Woods Hole, Massachusetts, United States of America; 3 Department of Medicine, Makerere University Medical School, Kampala, Uganda; 4 Department of Pediatrics and Child Health, Mulago Hospital, Makerere University Medical School, Kampala, Uganda; 5 Department of Botany, Canadian Institute for Advanced Research, University of British Columbia, Vancouver, British Columbia, Canada; 6 Department of Medicine, Albert Einstein College of Medicine, Bronx, New York, United States of America; Stanford University, United States of America

## Abstract

*Enterocytozoon bieneusi* is the most common microsporidian associated with human disease, particularly in the immunocompromised population. In the setting of HIV infection, it is associated with diarrhea and wasting syndrome. Like all microsporidia, *E. bieneusi* is an obligate, intracellular parasite, but unlike others, it is in direct contact with the host cell cytoplasm. Studies of *E. bieneusi* have been greatly limited due to the absence of genomic data and lack of a robust cultivation system. Here, we present the first large-scale genomic dataset for *E. bieneusi*. Approximately 3.86 Mb of unique sequence was generated by paired end Sanger sequencing, representing about 64% of the estimated 6 Mb genome. A total of 3,804 genes were identified in *E. bieneusi*, of which 1,702 encode proteins with assigned functions. Of these, 653 are homologs of *Encephalitozoon cuniculi* proteins. Only one *E. bieneusi* protein with assigned function had no *E. cuniculi* homolog. The shared proteins were, in general, evenly distributed among the functional categories, with the exception of a dearth of genes encoding proteins associated with pathways for fatty acid and core carbon metabolism. Short intergenic regions, high gene density, and shortened protein-coding sequences were observed in the *E. bieneusi* genome, all traits consistent with genomic compaction. Our findings suggest that *E. bieneusi* is a likely model for extreme genome reduction and host dependence.

## Author Summary

*Enterocytozoon bieneusi* is a clinically significant pathogen associated with human microsporidiosis, particularly in immunocompromised individuals. *E. bieneusi* is widespread in mammals, and there is no effective commercial treatment for infection. The pathogen cannot be readily cultivated, and animal models are limited. We therefore undertook a sequence survey and generated the first large-scale genomic dataset for *E. bieneusi*, which we used to study the organization and structure of its genome and to perform a comparative analysis with *Encephalitozoon cuniculi*, another microsporidian whose genome has been completely sequenced. The *E. bieneusi* genome showed many traits associated with genome compaction including high gene density, short intergenic regions, shortened proteins, and few introns. With one exception, all *E. bieneusi* proteins with assigned functions had *E. cuniculi* homologs. We found a paucity of genes encoding proteins associated with fatty acid and carbon metabolism. The possibility that these core functions are reduced in an intracellular parasite is intriguing, but because the genome sequence of *E. bieneusi* is incomplete, we cannot exclude the possibility that additional proteins associated with the various metabolic pathways would be discovered in a completed genome.

## Introduction

The microsporidia are a diverse group of obligate eukaryotic intracellular parasites that infect nearly all animal phyla (recently reviewed in [1],[2]) and are classified as Category B organisms on the NIAID Category A, B & C Priority Pathogens List. The first report of a microsporidian infection was over 150 years ago, when *Nosema bombycis*, a parasite of silkworms, was described. The phylum Microsporidia contains at least 1,200 species, divided into over 150 genera. Microsporidia are eukaryotes containing a nucleus with a nuclear envelope, an intracytoplasmic membrane system, and chromosome separation on mitotic spindles as well as Golgi. However, they lack canonical mitochondria and centrioles, possess prokaryotic size ribosomes (70S: consisting of a large subunit (LSU) rRNA (23S) and small subunit (SSU) rRNA (16S), and lack a discrete 5.8S rRNA [3]–[6]. The microsporidia were originally thought to be primitive protozoa, but were recently shown to be related to the Fungi, being either within or a sister group to this phylum (reviewed in [7]–[10]).

Microsporidiosis is considered a zoonotic and waterborne disease with agricultural consequence because it affects insects, livestock, wildlife, and domestic animals as well as humans. Fourteen microsporidian species are associated with human disease, but the majority of infections are caused by *Enterocytozoon bieneusi* [1], [10]–[15]. Clinical symptoms include chronic diarrhea, wasting and cholangitis. The majority of microsporidian infections in humans occur in immunocompromised patients, but occurrence in immunocompetent hosts is not unusual. Presently there is no effective commercial treatment for *E. bieneusi*-associated human microsporidiosis; although albendazole and fumagillin have been shown to be effective against *Encephalitozoon*-associated infections [16]–[20] and fumagillin has efficacy against *E. bieneusi* [21].

Although *E. bieneusi* is clinically the most significant microsporidium associated with human microsporidiosis, very little is known about this pathogen. It was first reported in 1985 [22], but progress towards the understanding of the biology of this organism has been hampered by the many challenges associated with working with *E. bieneusi*, including the difficulty in obtaining large quantities of purified *E. bieneusi* spores. *E. bieneusi* has also remained refractory to being reproducibly passaged in vitro, and when passage does occur, the yields are very low and inconsistent [23],[24]. As a consequence, much of the recent research on microsporidia has focused on the family Encephalitozoonidae, which has three members associated with human microsporidiosis, *Encephalitozoon intestinalis*, *Encephalitozoon hellem* and *Encephalitozoon cuniculi*. The reason is less for their clinical significance, but rather because they readily propagate in cell culture and in animals. The only microsporidian genome sequenced to date is *E. cuniculi*, with a genome size of 2.9 Mb, which is among the smallest eukaryotic genomes [25]. The *E. cuniculi* data revealed that its genome is highly compact; a total of 1,997 protein-coding sequences were identified, with an average intergenic region of 129 bases.

While much has been learned about microsporidia from the *E. cuniculi* genome project, *E*. *cuniculi* is not an adequate model for the study of *E. bieneusi,* which differs in a number of important characteristics. Specifically, ultrastructural examination of *E. bieneusi* in the biliary epithelium of rhesus macaques revealed (1) a lack of sporophorus vesicles or pansporoblastic membranes, (2) multiple rounded and elongated nuclei present within proliferative and sporogonial stages of the parasite, (3) late thickening of the sporogonial plasmodium plasmalemma, (4) presence of electron-translucent inclusions and electron-dense discs, and (5) direct contact of all stages with the host cytoplasm [26]. *E. bieneusi* was shown to abut the host-cell nucleic such that the nuclei are distorted and the parasite was seen in close association with the host mitochondria [26]. Significant clinical differences in sensitivity to albendazole distinguish these two microsporidia as well. Albendazole was shown to be effective against the Encephalitozoonidae, but not against *E. bieneusi*. The sequence of the *E. bieneusi* beta-tubulin gene has provided a molecular explanation for this difference in sensitivity [27]. These differences, along with the uncultivatability of *E. bieneusi* suggested that there would be differences between these two genomes. Thus, we undertook a genome sequence survey of *E. bieneusi* using recently developed purification methodology to obtain the necessary spores directly from infected humans. This sequence survey represents the first genomic sequence data available for this difficult-to-study organism. The aim of this project was to gain insight into the genomic architecture of this poorly understood microsporidian with respect to gene content and organization.

## Results/Discussion

### Genome Assembly and Composition

A significant challenge of this genome survey was obtaining a sufficient number of spores for library construction. With the absence of a robust in vitro cultivation method and the inability to produce enough spores in our rodent animal models, the only viable source was an infected human. Fecal samples from adult patients presenting with chronic watery diarrhea were screened by IFA and one patient with a very high *E. bieneusi* count was identified. Stool samples were collected, concentrated and purified using an extensive washing, filtration and centrifugation protocol (see Methods).

#### Genome assembly

**Figure 1:**
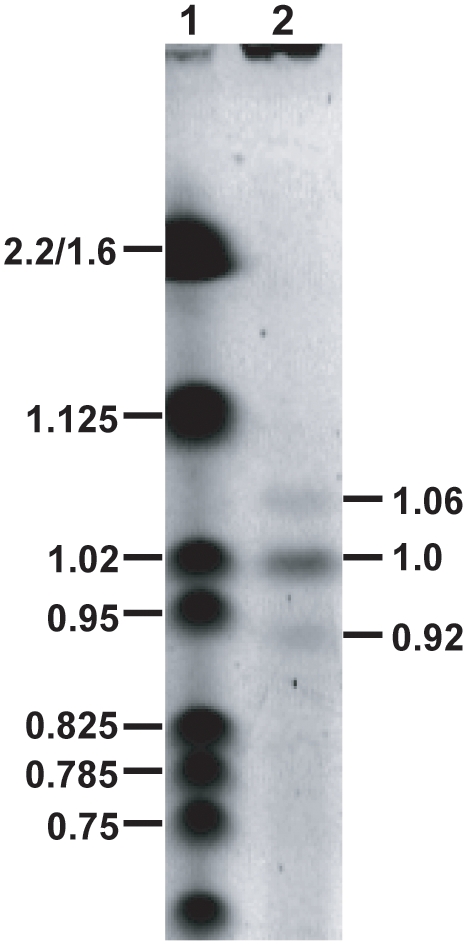
Estimation of the genome size of *E. bieneusi* based on pulsed field electrophoresis analysis. The *E. bieneusi* chromosomes were separated by electrophoresis (see Methods) and stained with ethidium bromide. The sizes of the chromosomal bands (lane 2; 0.92, 1.0, 1.06 Mb) were estimated using the MCID 3.0 software (Imaging Research Inc., St. Catharines, Canada). Based on densitometry analysis (ImageQuant TL 1-D analysis software; GE Healthcare Bio-Sciences Corp., Piscataway, NJ), the ratios of the bands were estimated to be 1∶4∶1. Using these ratios, the genome was estimated to be ∼6 Mb. *S. cerevisiae* chromosomal size standards (Bio-Rad) were included (lane 1) and their sizes (Mb) are shown.

The genome size of *E. bieneusi* was estimated by pulsed field electrophoresis analysis (Figure 1). Three chromosomal bands were observed with estimated molecular weights of 0.92, 1.0 and 1.06 Mb. The ratio of the band intensities was estimated to be 1∶4∶1; thus predicting a genome of 6 Mb. This is an overestimate if the 1.0 Mb band represents fewer than four chromosomes.

A random genomic library of 2–3 kb inserts of *E. bieneusi* DNA was constructed and 53,268 reads were generated. After filtering out bacterial contamination (see Methods) 34,212 reads (27.4 million bases) were assembled into 1,742 contiguously assembled segments (contigs), using the Paracel GenomeAssembler with default program parameters and quality scores (Agencourt Bioscience Corporation, Beverly, MA). The combined contig length was 3.86 Mb. Assuming a genome of 6 Mb, the number of bases (3.86 Mb) contained within the 1,742 contigs represents about 64% of the genome.

Gene predictions and automated annotations were generated by Integrated Genomics (Chicago, IL) using the ERGO genome analysis suite [28] and FunGene [29]. We additionally used Glimmer3 [30] to identify additional candidate genes not called by ERGO and FunGene. These datasets, as well as the contig sequences, were searched using BLAST (blastp, tblastn; [31],[32]) to identify homologs of *E. cuniculi* and *Saccharomyces cerevisiae* genes. Also, we queried Genbank's non-redundant protein database using blastx to identify similarity to other known proteins. The final gene complement contained 3,632 protein-coding genes, 74 tRNA genes and 98 ribosomal RNA genes (5S, SSU and LSU rRNAs) (Table 1). The *E. bieneusi* genome survey data have been deposited at DDB/EMBL/GenBank and are also available at the Biodefense and Public Health Database (BioHealthBase) Bioinformatics Resource Center website (http://www.biohealthbase.org).

**Table 1:** *E. bieneusi* statistics with a comparison to the *E. cuniculi* genome.

	*E. bieneusi*	*E. cuniculi* [25]
Genome Size, Mb	6^1^	2.9
Chromosome Number	6^1^	11
Scaffolds	1,646	11
Scaffold N50 bp	[2,349]	NA
Contigs	[1,742]	NA
Contig N50 bp	[1,977]	NA
Assembled Mb	[3.86]	2.5
Sequence Coverage, %	[64]	86
G+C Content, %	[25^2^]	47
Gene Models	[3,804]	2,063
Gene Density	[1/1,148 bases^2^]	1/1,025 bases
No. of SSU-LSU rRNA Genes	Unknown^3^	22
No. of 5S rRNA Genes	Unknown^3^	3
No. of tRNAs	[46]	46
No. and Sizes of tRNA Introns	[2 (13, 30 bp)]	2 (16, 42 bp)
No. of tRNA Synthetases	[21]	19
No. and Sizes of Splicesomal Introns	[19 (36–306 bp)]	13 (23–52 bp)
Predicted CDS^4^	[3,632]	1,997
Mean Intergenic Region, bp	[127^2^]	129
Median CDS, bp	[579]	858
Mean Size of CDS, bp	[995^2^]	1,077
Overlapping CDS?	Yes	Yes
No. of CDS Assigned to Functional Categories	[653 (39%)^5^]	884 (44%)

1 Estimation based on PFGE data.

2 Values based on analysis of contigs >5 kb and their encoded genes. Bracketed values indicate values based on the survey data and not a complete genome.

3 The 5S, 5.8S-SSU and LSU rRNA genes have been identified in *E. bieneusi* but are located on short contigs, with very short regions of sequence upstream and downstream, suggesting that these are either surrounded by sequences that are difficult to clone into *E. coli* or are difficult to sequence, such as highly repeated sequences. Therefore, the copy number of these genes cannot be determined at this time.

4 CDS, coding sequences; NA, not applicable

5 Values based on data from Table S4.

#### Ribosomal RNA (rRNA) genes

Approximately 124 contigs (all except one were under 2 kb) showing high similarity to the *E. bieneusi* ribosomal RNA gene sequences deposited in GenBank were further assembled into nine contigs using Sequencher (GeneCodes Corp., Ann Arbor, MI) with the largest contig being 4.6 kb. At least one contig contained a partial 5.8S rRNA fused to the LSU rRNA, intergenic region and part of the SSU rRNA genes. Several contigs contained a partial sequence of the SSU rRNA gene and an intergenic region upstream of this gene. The apparent fusion of the 5.8S and LSU rRNA genes in *E. bieneusi* was similar to that observed in *E*. *cuniculi* [25]. Analysis of these sequences suggested at least two different SSU rRNA genes were present in the *E. bieneusi* genome. The observation that none of the *E. bieneusi* contigs containing the rRNA genes were located on contigs greater than 5 kb suggests that the ribosomal RNA genes were either adjacent to repetitive sequences (limiting their assembly) or these regions were unclonable in *E. coli*. Sequences adjacent to the *E. bieneusi* ribosomal genes were very short and as a result, determination of the genomic context and thus, the number of ribosomal genes was not possible. In the *E. cuniculi* genome, twenty-two SSU-LSU rRNA genes were identified, with two genes located per chromosome in the subtelomeric region [25],[33]. In addition, three 5S rRNA genes were found on three different chromosomes. The regions upstream of the SSU ribosomal genes in *E. cuniculi* are highly repetitive [25],[33],[34]. Our data are consistent with a similar rRNA gene organization in the *E. bieneusi* genome.

#### Transfer RNAs (tRNAs) and codon usage

Forty-six unique tRNA genes were identified using tRNAscan-SE [35] to analyze the *E. bieneusi* genomic survey sequences. The same number of tRNAs was annotated in *E. cuniculi* ([25]; Biodefense and Public Health Database). This number is comparable to the tRNA complement of several parasitic protozoa, including *Cryptosporidium parvum* (45 tRNAs; [36]), *C. hominis* (45; [37]), *Theileria parva* and *T. annulata* (47 tRNAs; [38],[39]), and *Plasmodium falciparum* (43 tRNAs; [40]). While there was only a single tRNA gene for most anticodons, three tRNA genes were found for tRNA^Lys^ (UUU), and tRNA^Asn^ (GUU). The tRNA genes were located on 25 contigs, suggesting they were spread throughout the *E. bieneusi* genome rather than clustered. However, some “loose” tRNA gene clustering was observed. Eight of the identified tRNA genes were located within a 67.6 kb region on contig C168. On the other hand, only two tRNA genes were found within about 84 kb on contig C678. The two tRNA^Met^ genes were found within a 1.3 kb region on contig C365 and were the only tRNA genes located on this contig, which was greater than 37 kb. Five tRNA genes were located on contig C30 (7.7 kb), of which two tRNAs (tRNA^Thr^ (CGU) and tRNA^Ser^ (CGA)) were separated by only 18 nucleotides.

The *E. bieneusi* tRNA genes showed codon bias because of the genome's unusually high A+T content, with preferential use of one synonymous codon over the other(s). All A- or U-containing codons were used more frequently than G- or C-containing synonyms. This bias ranged from about 3.5- to 12-fold for leucine. One extreme example was the codon usage of isoleucine, where ATA and ATT combined accounted for 92% (37.4 and 55%, respectively) and ATC only accounted for 7.6% of isoleucine codons. In many unicellular organisms, invertebrates, and plants, codon biases result from a co-evolution between codon usage and tRNA abundance as a means to optimize the efficiency of protein synthesis [41]–[43]. Also, tRNA abundance appeared to be roughly correlated with tRNA gene copy number. In the *E. bieneusi* genome, there was a weak correlation between codon frequency and tRNA gene copy number since all of the multi-gene tRNAs decoded the high abundant codons. For example, the three tRNA^Lys^ genes all decoded the codon AAA, which was found more than 800 times in every 10,000 codons, while one tRNA^Lys^ gene decoded the codon AAG, which had a frequency of 150 per 10,000 codons.

#### Gene duplication

Nineteen protein-coding genes with multiple copies within the *E. bieneusi* genome were identified on twenty contigs, of which seventeen are contigs, greater than 11 kb (Table S1). In the *E. cuniculi* genome, these genes are single-copy genes, with the exception of the gene encoding serine hydroxymethyltransferase. Most of these multi-copy genes in *E. bieneusi* encoded enzymes or proteins with important cellular functions and the majority were located on different contigs. One exception was three genes encoding the ER lumen protein retaining receptor (KDEL receptor 2; EBI_25452, EBI_21804 and EBI_25453), which were tandemly located on contig C1044 and separated by intergenic lengths of 171 and 28 nucleotides. In contrast, six *E. bieneusi* genes encoding the proteasome regulatory subunit YTA6 of the AAA family of ATPase were located on three contigs, with three genes (EBI_22585, EBI_25889 and EBI_25890) adjacently located on contig C154, one gene (EBI_26042) on contig C166 and two genes (EBI_22798 and EBI_22799) on contig C167. Interestingly, another gene (EBI_22558), encoding a homolog to a second *E. cuniculi* proteasome regulatory subunit YTA6 of the AAA family of ATPase, was located ∼4 kb from the three genes on contig C154. Comparison of the amino acid sequences of the six homologs showed that homologs on the same contig were more similar to each other than to homologs on the other two contigs (data not shown).

On contigs C167 and C1951, there were four multi-copy genes that encoded a cation-transporting ATPase, a protein with similarity to the chloroplast 28 kDa ribonucleoprotein, a hypothetical protein homologous to ECU04_1510, and a protein with similarity to the suppressor of stem loop protein 1 (Figure S1). On contigs C167 and C1951, the genes encoding the 28 kDa ribonucleoprotein (EBI_26075 and EBI_26413, respectively) and hypothetical protein ECU04_1510 (EBI_26076 and EBI_23251, respectively) were identical at the amino acid level and were located within ∼2 kb regions that were nearly identical at the nucleotide level. The gene encoding cation-transporting ATPase located on contig C1951 (EBI_26421) had an identity of 81% and 80% to the genes located on contig C166 (EBI_26064) and C167 (EBI_26069), respectively. The suppressor of stem loop protein 1 encoded on contig C1951 (EBI_26368) contained a 160 amino acid truncation at its amino-terminus compared to the homologs encoded on contigs C167 (EBI_26095) and C168 (EBI_22830).

### Evidence for a Compact *E. bieneusi* Genome

#### Intergenic regions

Analysis of the 46 contigs greater than 5 kb showed that the *E. bieneusi* genome was compact, with the intergenic regions ranging in length from −24 to 1,876 bases, with a mean length of 127 bases (Table 1). The lengths of the intergenic regions in *E. bieneusi* were similar to those of *E. cuniculi*, in which the mean length of the intergenic regions is 129 bases. The mean gene density of the *E. bieneusi* genome was estimated to be 1 protein-coding gene/1,148 bases, comparable to that observed in *E. cuniculi* (1 protein-coding gene/1,025 bases).

#### Overlapping genes

Several putative overlapping protein-coding genes were identified in *E. bieneusi*. Four sets of overlapping genes (EBI_22332/EBI_25749; EBI_26851/EBI_26852; EBI_26372/EBI_26373; and EBI_23221/EBI_23245) had overlapping stop codons (≤4 base overlap) and three sets (EBI_21963/EBI_25571, EBI_22813/EBI_26114 and EBI_23264/EBI_26462) overlapped by 24, 15 and 13 bases, respectively. With the exception of EBI_23264/EBI_26462, the overlapping genes in the other five sets were oriented in opposite directions. Two other potential overlapping sets of ORFs (EBI_24933/EBI_27528 and EBI_25987/EBI_22714) were on opposite strands of their respective contigs and had overlapping start codons in different reading frames. Of these nine sets of overlapping genes, only one gene set (EBI_21963/EBI_25571) encoded proteins with assigned functions; a second set (EBI_23221/EBI_23245) included a gene encoding a conserved uncharacterized protein; and the remaining seven gene sets included at least one hypothetical protein. In *E. cuniculi*, no overlapping genes encoding proteins with assigned functions were reported [25],[33]. However, examination of the *E. cuniculi* gene annotation does identify several overlapping genes (e.g. ECU05_1230/ECU1240 and ECU08_1090/ECU08_1100).

#### Introns

DNA sequences of contigs greater than 10 kb were analyzed by FGENESH (http://www.softberry.com; [44]), an HMM-based gene structure prediction program, to identify putative introns. For the FGENESH analysis, the search parameters were set for *Schizosaccharomyces pombe*. We also used the splice-aware aligner Exonerate (http://www.ebi.ac.uk/~guy/exonerate/; [45]) to search the contigs with both *E. cuniculi* and *S. cerevisiae* proteins. When Exonerate identified a region with a possible intron (46 instances), we called the gene using GeneWise (http://www.ebi.ac.uk/Tools/Wise2/; [46]). The Exonerate/GeneWise search predicted nineteen possible introns in eighteen genes, all with canonical splice sites and all of phase 0 type. Genes with putative introns were translated and aligned to several homologs using Clustal [47] to evaluate their validity (Figure S2). Because of low sequence identity or potential species-related insertions, the introns could not be validated for most of these genes. For example, the alignment of one protein (EBI_22563) showed the full length translation aligned as well or better than the spliced version (Figure S2A). The alignment of a second protein (EBI_21989; Figure S2B) suggested the spliced form aligned better than the unspliced version, but there were relatively few homologous proteins available for comparison. The alignment of a third protein showed an insertion relative to many taxa (EBI_22717; Figure S2C), but a similar insertion was evident in the *E. cuniculi* homolog. Whether any of these candidates or other *E. bieneusi* genes contain introns could not be experimentally confirmed by standard cDNA sequencing since *E. bieneusi* is uncultivable. Nevertheless, *E. bieneusi* introns appear to be a rare occurrence at best.

Introns are also rare in *E. cuniculi*; a single putative splicesomal intron was found in each of eleven ribosomal protein genes, two were found in the CDP-diacyl-glycerol serine phosphatidyltransferase gene [25],[33] and another in the *Sec*61 gene [48]. In the *E. bieneusi* genome data, we identified eight of these eleven ribosomal proteins where introns were found in *E. cuniculi,* but none were interrupted by splicesomal introns in *E. bieneusi*. We did not identify homologs of the remaining three ribosomal proteins nor of the CDP-diacyl-glycerol serine phosphatidyltransferase gene, based on protein alignments. Another indirect indication that introns were present in the genome was the presence of the splicesomal machinery. Many components of the spliceosome were identified in the *E. cuniculi* genome, including genes for U2 and U6 snRNAs, and genes for several snRNP-associated proteins [25]. We did not find evidence for any snRNA genes in our survey, but did identify homologs of a small number of U3 and U6 sn-RNA-associated proteins. The presence of introns cannot be ruled out without a complete genome, but the paucity of genes for splicesomal components, the absence of detectable introns in the current survey, and specifically the absence of introns from the eight genes where they have been found in *E. cuniculi*, all suggest that introns, if present, are less common than in the *E. cuniculi* genome. Two *E. cuniculi* tRNA genes contain tRNA introns. We found both tRNA genes in *E. bieneusi* and both contained introns at the same positions.

#### Analysis of the lengths of *E. bieneusi* proteins

**Figure 2:**
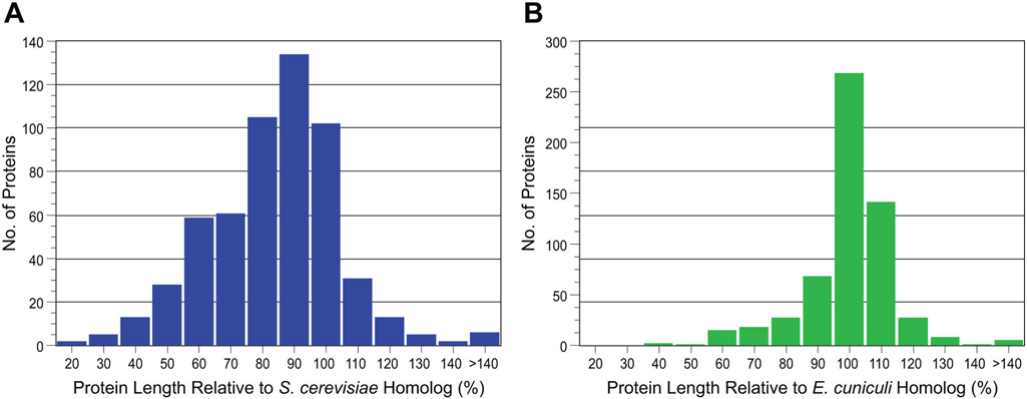
Comparison of the lengths of *E. bieneusi* proteins with their respective *S. cerevisiae* (*Sc*) and *E. cuniculi* (*Ec*) homologs. Only *E. bieneusi* proteins with assigned functions that were located on the larger contigs were included in these comparisons. (A). Lengths of the *E. bieneusi* proteins (n = 566) relative to their *S. cerevisiae* homologs, expressed as a percentage. (B). Lengths of the *E. bieneusi* proteins (n = 580) relative to their *E. cuniculi* homologs, expressed as a percentage. *E. bieneusi* proteins that were shorter or larger than their respective homologs have percentages less than 100% or greater than 100%, respectively.

Based on the genomic data, the mean size of the *E. bieneusi* coding sequences was very close to that of *E*. *cuniculi* (Table 1). The lengths of *E*. *bieneusi* proteins with assigned functions were compared to either their *S. cerevisiae* or *E. cuniculi* homologs to determine if these comparisons were consistent with genome compaction (Figure 2). The lengths of 566 *E. bieneusi* proteins were compared to their *S. cerevisiae* homologs and of these, 505 (89.2%) were on average 25% shorter (Figure 2A). Fifty-seven (10.1%) of the proteins were on average 16% longer than their respective *S. cerevisiae* homologs and the remaining four (0.7%) were the same length. Overall, the *E. bieneusi* proteins were 21% shorter (mean relative size difference). The next analysis compared the protein lengths of *E. bieneusi* and *E. cuniculi* homologs (Figure 2B). A total of 580 homologs were examined and of these, 386 (66.6%) *E. bieneusi* proteins were on average 11% shorter, 182 (31.4%) were on average 8% longer and 12 (2%) were the same length. Overall, the *E. bieneusi* proteins were 5% shorter (mean relative size difference) suggesting that *E. bieneusi* proteins were even more compact than those of *E. cuniculi*. A comparison of 559 *E. cuniculi* proteins to their respective *S. cerevisiae* homologs revealed 469 (83.9%) proteins were on average 22% shorter, 80 (14.3%) were on average 14% longer and 10 (1.8%) were the same length, with a mean relative size difference of 16%. Our *E. cuniculi*-*S. cerevisiae* comparison was consistent with the data of Katinka et al. [25]. Vivarés et al. [49] postulated that truncation of the *E. cuniculi* genes is associated with a decrease in the number of protein-protein interactions, as a result of decreasing complexity of the proteome.

To further support the general observation that *E. bieneusi* proteins are compact, a comparison of the lengths of fourteen homologs from different organisms was made (Tables S2 and S3). The lengths of eight subunits of the T-complex protein 1 were compared in twelve eukaryotic organisms (Table S2). The *E. bieneusi* homologs were the shortest for four of these homologs and were among the shortest for the remaining four, after their *E. cuniculi* homolog. A similar comparison was made for five DNA-directed RNA polymerase III subunits and for the transcription initiation factor TFIII B subunit (Table S3). These data show that the *E. bieneusi* homologs are either the shortest, or one of the shortest homologs of each protein observed, thereby supporting the notion of genome compaction in this parasite.

### Functional Categories of the *E. bieneusi* Genes

1,702 *E. bieneusi* genes were assigned functions using the ERGO annotation pipeline, information from BLAST searches, and the KEGG automated annotation server [50]. 1,199 of the *E. bieneusi* genes with assigned functions were homologs of one of the 884 *E. cuniculi* proteins in eleven functional categories (Table S4). Functional domains were detected in an additional 111 proteins annotated by ERGO as “hypothetical” and these proteins are included in Table S4. Only one gene with assigned function, methionine adenosyltransferase 1, was found in *E. bieneusi*, but not *E. cuniculi*. The remaining proteins (∼615) showed either similarity to an *E. cuniculi* hypothetical or functionally undefined protein, no similarity to any protein in GenBank, high similarity to a bacterial protein, or were very short in length.

**Figure 3:**
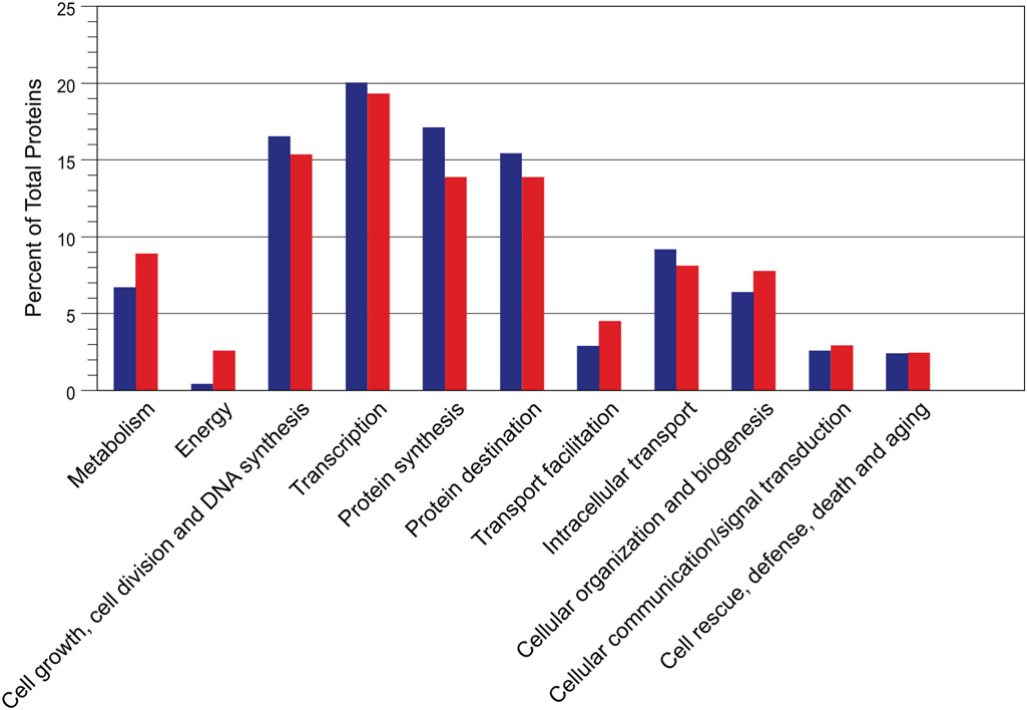
Grouped bar graph showing the distribution percentages of *E. bieneusi* (blue) and *E. cuniculi* (red) proteins among the functional categories. *E. bieneusi* proteins (653 proteins) were assigned to one of eleven functional categories listed in Katinka et al. [25]. For comparison, the distribution percentages of the *E. cuniculi* proteins (884 proteins) were included [25]. The corresponding gene lists for both organisms are presented in Table S4.

The protein-coding DNA sequences of known function were assigned to one of eleven functional categories (Figure 3 and Table S4) and from the distribution of these genes, a view of the parasite's lifestyle can be glimpsed. With the caveat that not all protein-coding genes have been identified in *E. bieneusi*, approximately 37% of the identified genes were associated with the replication of the parasite. A roughly equivalent number of genes had functions related to protein synthesis and trafficking. A significantly lower number of proteins were associated with lipid, fatty acid and isoprenoid metabolism, and energy generation functions (0.7 and 0.5%, respectively), but the moderate number of genes associated with transport of products (9%), was in line with the obligate intracellular lifestyle of *E*. *bieneusi* and its dependence on the host for most of its needed nutrients.

Given the possibility that only 64% of the *E. bieneusi* genome had been sampled, one cannot conclude anything from the absence of any one gene, but analyzing entire pathways or classes of genes hints at the overall makeup of the genome. Indeed, a comparative analysis of the protein repertoire of *E. bieneusi* and *E. cuniculi* showed that many functional categories are most likely similarly represented in the genome but others may not be. Not surprisingly, a large proportion of the *E. cuniculi* protein-coding genes related to DNA replication, transcription, translation, protein folding, trafficking, and degradation were also identified in the *E. bieneusi* survey (Figure 3 and Table S4), suggesting these functional classes are similarly represented in the two genomes. In contrast, however, a surprising number of genes related to energy generation, lipid, fatty acid and isoprenoid metabolism and mRNA splicing found in *E. cuniculi* are absent from the *E. bieneusi* survey. In particular, genes encoding proteins for core carbon metabolism are surprisingly rare with only three of the twenty-three *E*. *cuniculi* proteins with functions in glycolysis, trehalose metabolism, and the pentose phosphate pathway identified in *E*. *bieneusi*. The absence of trehalose metabolism has particular implications for microsporidia because it has been suggested to play several roles in this lineage, including (1) rapid increase of the osmotic pressure within the spores required for polar tube extrusion upon hydrolysis of trehalose; (2) protection against freezing or desiccation; and (3) use as a potential energy source [51],[52]. Since this pathway contains relatively few genes, its apparent absence in the survey may simply be a reflection of incomplete coverage.

It is possible that the absence of genes associated with energy generation and lipid metabolism is due to sampling bias, but it is very difficult to imagine what could drive such a bias. The current draft sequence read coverage is >27 Mb, or well over 4X. If the genome size is close to the estimated 6 Mb and coverage is random, we would expect to see a combined contig length of approximately 6 Mb: the probability of sequencing any base follows a Poisson distribution and at 4X coverage, the probability that any one base has been sampled at least once is >98%. However, the draft sequence is equal to only ∼64% coverage and many contigs are over-tiled. This suggests the quantity of unique genome sequence is lower (3–4 Mb). Even if we assume that coverage is not random and that we have completely missed sequencing 2 Mb of the genome, it is improbable that ten or more genes from any one pathway are clustered in the missing sequence. The relatively even sampling of other gene classes represented in the *E. cuniculi* genome (Figure 3 and Table S4), suggests instead that these categories are underrepresented in the *E*. *bieneusi* genome. Microsporidia are known to rely extensively on their hosts for energy and nutrients, and it seems likely this is also true of *E. bieneusi*. Nevertheless, a parasite with no obvious means of deriving energy from sugars would represent an extreme form of reduction and host-dependence, a possibility that needs further investigation.

### Summary

The inability to cultivate a eukaryote is a serious impediment to characterizing its whole nuclear genome. Many parasites that form intimate associations with their hosts are often uncultivable. *E. bieneusi* is one such intracellular parasite, so to initiate a complete characterization of its genome, we generated ∼34,000 genome survey sequences, amounting to ∼3.86 Mb of unique sequence using material directly isolated from infected humans. The genome size is estimated to be 6 Mb based on the pulse field electrophoresis data. However, this may be an over-estimation since this is based on the accuracy of the calculation of the chromosomal band intensities. Indeed, comparing our genomic data to the genome of *E. cuniculi* supports the conclusion that there is significantly less than 6 Mb of unique sequence in the *E. bieneusi* genome. Specifically, the identification of 21 tRNA synthetases and 46 tRNA genes, the low number of *E. bieneusi*-specific protein-coding genes, as well as over-representation of reads within certain contigs, all suggest a high proportion of the unique sequence in the genome has been sampled. Though the genome size of *E. bieneusi* is over twice as large as *E. cuniculi*, a similar gene density is observed. This inconsistence between genome size and genome content could be explained if the *E. bieneusi* genome has chromosome heterogeneity, in particular partial duplications. Duplicate gene copies found in our *E. bieneusi* survey support this. Moreover, evidence for chromosome heterogeneity in two microsporidia has been reported. Molecular karyotyping analysis of *Paranosema grylli* identified chromosomal heterogeneity resulting from chromosomal rearrangements [53]. The *Brachiola algerae* survey showed a discrepancy between its predicted coding capacity and the finding that no recognizable genes were found that were not present in other microsporidian genomes [54].

Nonetheless, assuming a genome size of 6 Mb, the survey sequences represent at least 64% of the genome, a significant advance for the study of *E. bieneusi* since only a few genes were available prior to this survey. In addition, with these data, microarray and RT-PCR experiments for identification of genes involved with host-pathogen interactions, infection, as well as identification of potential therapeutic drug targets can be initiated, since the limitations on cultivation are substantially mitigated by the prior knowledge of many gene sequences. Only 47% of the protein-coding ORFs were assigned function, suggesting that many of the unassigned ORFs may represent novel genes. Future efforts focused on studying these proteins may identify novel proteins associated with the obligate lifestyle of *E. bieneusi*.

This genome sequence survey demonstrates the feasibility of a complete genome analysis of *E. bieneusi*, despite the lack of cultivation, and points to many biologically interesting questions that would be addressed by the complete sequence. In particular, our survey suggests that a comprehensive comparison of gene complements with *E. cuniculi* and to other microsporidian genomes will be of tremendous interest. The severe reduction and perhaps even absence of intact pathways for core carbon metabolism is unexpected and suggestive of a unique level of host dependence; in turn suggesting many novel mechanisms for interactions with the host (perhaps associated with the many proteins with unknown functions). Similarly, a number of proteins related to infection were not identified. The absence of these proteins and pathways will need to be tested using independent evidence, or the exact nature of the genome structure determined unambiguously. Nevertheless, the genome survey of *E. bieneusi* presented here provides the first available genomic data that will add to the growing knowledge base for understanding this organism, which should result in the development of diagnostic tools and potential therapeutics, and also highlights *E. bieneusi* as a potential model for extreme reduction and host dependence.

## Methods

### Source of *E. bieneusi* Spores

Stools were collected from adult HIV/AIDS patients admitted to the Mulago Hospital, Kampala, Uganda with chronic watery diarrhea. Stool samples were tested for *E. bieneusi* by immunofluorescence assay (IFA) using *E. bieneusi*-specific polyclonal antibodies [55]. Fecal samples from patients who were *E. bieneusi*-positive were collected, concentrated by centrifugation (4,000×g; 40 minutes) and resuspended in phosphate-buffered saline (PBS). The concentrated fecal samples were stored at 4°C until they were shipped to Tufts University for purification and analysis.

### Purification of *E. bieneusi* Spores

*E. bieneusi* spores were purified from fresh stools of infected adult humans using the method described by Zhang et al. [56]. Briefly, the concentrated feces were homogenized and serially filtered through American Standard sieves of decreasing pore size (425, 180, 100 and 63 µm; Newark Wire Cloth Company, Newark, NJ). The spores were centrifuged at 3,200g for 40 minutes and the spore pellet was washed 4 times with distilled water (3,200g, 20 minutes). The pellet was resuspended, mixed with saturated sodium chloride and centrifuged at 1,000g for 15 minutes. The spores remained resuspended in the salt solution (density ∼1.2 g/cm^3^), separated from the bacteria and particulate matter, which either settled on top of the salt solution or sedimented to the bottom of the tube. The salt solution was carefully removed with a needle and syringe, and the spores collected, washed and resuspended in PBS. The spores were further purified by sequential centrifugation through 72% isotonic Percoll, 30–60% (w/w) sucrose gradient, and 10–50% (w/v) preformed iodixanol gradient prepared from OptiPrep density gradient medium (Sigma-Aldrich Co., St. Louis, MO). Purified spores were stored at 4°C.

Spores purified from one patient (isolate 348) were used for the genome survey. The first spore preparation (∼2.2×10^9^ in 1.6 ml sterile PBS) was checked for bacterial and fungal contamination by plating an aliquot (10 µl/plate) on blood agar and Saboraud plates and the plates incubated at 37°C or 30°C, respectively. The plates were visually checked daily for contamination for 3 days. Approximately 200–300 colonies and 50 colonies were observed on the blood agar and Saboraud plates, respectively. The bacterial and fungal contamination was estimated to be <0.002%. A second purified spore preparation (∼2×10^9^ spores) from the same patient was performed using the same protocol (<0.05% bacterial and fungal contamination).

### Extraction of Genomic DNA

Genomic DNA was purified using a modified Proteinase K-phenol extraction protocol [57],[58]. The Proteinase K, RNase and linear acrylamide solutions used in the extraction protocol were specifically selected because they were certified by the vendor to have undetectable levels of bacterial DNA and RNA. Spores (∼2.2×10^9^) were collected by centrifugation (15,000×g; 4 minutes) and resuspended in 400 µl of lysis buffer (100 mM EDTA, pH 8.0, 0.2% sodium deoxycholate, 1% sodium lauryl sarcosine). Proteinase K (catalog no. 03115887001; Roche Diagnostics Corp., Indianapolis, IN) was added to the sample. The spores were subjected to 5 freeze-thaw cycles, then incubated at 50°C for 2 days. The spores were again subjected to 2 freeze-thaw cycles, RNase (2 µl, 10 µg/µl; catalog no. 11119915001; Roche) was added, and the sample incubated at 37°C for 30 minutes. Proteinase K (10 µl; Roche) was added to the sample and 2 additional freeze-thaw cycles were performed before the sample was incubated at 50°C for another 2 days. An equal volume of phenol:chloroform:isoamyl alcohol (25∶24∶1) was added and the sample was mixed on a rotator for 2 hours at room temperature. The sample was centrifuged at 15,000×g for 7 minutes. The aqueous phase was removed and transferred to a new tube using a wide-bore pipet tip. The DNA was precipitated by the addition of 3 M sodium acetate, pH 5.2 (45 µl), linear acrylamide (3 µl; Ambion) and 100% ethanol (1 ml) and left at −80°C overnight. The DNA was collected by centrifugation (15,000×g; 15 minutes), the DNA pellet washed with 100% ethanol, and dried briefly. The DNA was resuspended in 50 µl TE and its concentration determined using Quant-IT™ PicoGreen® dsDNA Assay Kit (Invitrogen Corp., Carlsbad, CA). The estimated yield was 2.5 µg from 1.5×10^9^ spores. A 100 ng aliquot of the final genomic DNA preparation was run on a 0.7% TAE gel and also a portion of the β-tubulin gene was amplified using *E. bieneusi*-specific primers for quality control purposes. A second genomic DNA preparation was performed using the 2×10^9^ spores from the second purified spore preparation. The DNA yield of the second genomic preparation was 2.2 µg.

### Pulsed Field Electrophoresis

Agarose plugs containing 10^8^ purified *E. bieneusi* spores were prepared with 1% low melting agarose (Bio-Rad, Richmond, CA). The plugs were immersed in 5 ml lysis buffer (0.5 M EDTA, pH 8.0, 1% N-lauroylsarcosine, 5 mg/ml Proteinase K; Sigma Chemical Co., St. Louis, MO) and incubated for 72 hours at 50°C [57]. The plugs were placed into the PFGE wells of a 1% agarose gel in 0.5×TBE buffer (45 mM Tris, 45 mM boric acid, 1 mM EDTA) and electrophoresis was performed in a contour-clamped homogenous electrical field (CHEF DR III System, Bio-Rad). The gel was run with a pulse time of 90 to 200 second switches at 100 V for 72 hours at 14°C. A *S. cerevisiae* chromosomal size standard (Bio-Rad) was included on the gel. Two independent runs were performed.

### Library Construction and Validation

Using the two genomic DNA preparations, two genomic libraries with random 2–3 kb inserts were constructed by Agencourt Bioscience Corporation in their proprietary high copy number vector. Genomic DNA was hydrodynamically sheared in the Hydroshear (GeneMachines, San Carlos, CA) and separated on agarose gel. A fraction corresponding to ∼3,500 bp was excised from the gel and purified by the GeneClean procedure (Qbiogene, Morgan Irvine, CA). The purified DNA fragments were blunt-ended using T4 DNA polymerase and ligated to unique BstXI-linker adapters. These linkers are complementary to an Agencourt-developed high copy vector cloning site, while the overhang is not self-complementary. Therefore, the linkers will not concatemerize nor will the cut-vector readily religate to itself. The linker-adapted inserts were separated from the unincorporated linkers on a 1% agarose gel and again purified using GeneClean. Products were ligated to BstXI-cut vector to construct a “shotgun” subclone library. Ligation products were used to transform ElectroMAX DH10B cells (Invitrogen), plated onto agar containing kanamycin and incubated overnight at 37°C. Transformants were picked for sequencing and liquid cultures were grown overnight at 37°C. DNA was purified using Agencourt's proprietary large-scale automated template purification systems using solid-phase reversible immobilization or SPRI.

The quality of the first library was evaluated by sequencing 192 clones. Sequences were compared against GenBank's nonredundant protein database at the National Center for Biotechnology Information (NCBI; http://www.ncbi.nlm.nih.gov/Genbank) using the BLASTX algorithm [31],[32]. Only the top BLAST matches were used to classify the reads as either eukaryotic or bacterial. Approximately 17% of the sequences had highest identity to bacteria, which was higher than expected based on the assay for bacterial contamination. Thirty-three percent of the sequences showed highest similarity to *E. cuniculi* and 50% showed no significant homology to either eukaryotic or bacterial sequences. Although the proportion of bacterial sequences was high, we decided to proceed with this library since a new preparation of spores was not likely to be of higher quality. However, not enough clones were obtained from this library to complete the genome survey and a second 2–3 kb library was constructed using the genomic DNA from the second preparation. The quality of this library was evaluated and was estimated to have <5% bacterial sequences.

### Sanger Sequencing of the Libraries

The purified DNA samples were sequenced using ABI 3.1 BigDye terminator chemistry on ABI 3730XL automated sequencers (Applied Biosystems, Foster City, CA) by Agencourt Bioscience Corporation. Sequence data were transferred to Linux machines for processing. Base calls and quality scores were determined using the program PHRED [59],[60]. Average high-quality read length (Phred20) was 802 bases. Reads were assembled by Agencourt using Paracel, with default settings. Reads from both libraries were treated as a single dataset.

### Identification of Bacterial Contamination

Since validation of the genomic library showed the presence of bacterial sequences (multiple taxa), the contigs were classified as from either *E. bieneusi* or bacteria based on BLAST analysis results. The nucleotide sequence of each contig was used to query the NCBI non-redundant nucleotide database using the BLASTN algorithm [31],[32]. The percent AT content of each contig was determined and an obvious biphasic curve was observed. Visual examination of these data indicated that contigs with significant matches (Expect (E) <e^−80^) to *Pseudomonas* had an AT content of 30–49%, while sequences with significant matches to *E. cuniculi* had an AT content of 60–79%. The majority of contigs (>90%) with an E value <e^−5^ match to a non-bacterial sequence had an AT content of ≥50%. Based on this observation, bacterial contigs were identified as those contigs whose highest sequence identity was to a bacterial sequence with an E value <e^−50^ and an A+T content of <50%. A total of 1,079 contigs met both of these criteria and were removed from the final dataset. This represents 38.1% of the number of contigs, 32.1% of the number of bases in the contigs, but only 9% of the total number of bases assembled into contigs. The largest bacterial contig was 8,147 bases and the average length of bacterial contigs was 1,689 bases. The majority of the bacterial contigs (77.7%) were less than 2 kb in length. Approximately 86% (927 contigs) of these contigs had highest similarity to *Pseudomonas*.

Based on these criteria, 1,742 contigs assembled from 34,212 reads, remained in the *E. bieneusi* genome survey and represent 3.86 Mb. Significantly, the 38 largest contigs of the original dataset were *E. bieneusi* and ranged in length from 8,405 to 131,639 bases (Table S5). Of the 1,742 contigs, 4 were greater than 100 kb in length and 136 were greater than 2 kb. Over 90% of the contigs were less than 2 kb in length. The total number of bases represented by contigs greater than 2 kb was 1.91 Mb and 1.95 Mb were represented by contigs less than 2 kb. The *E. bieneusi* genome has an overall A+T content of 75% based on the 49 contigs of greater than 4 kb in length (Table S5). The A+T content of the individual contigs within this group ranged from 59% to 80%. The overall A+T content decreased to less than 62% for contigs smaller than 2 kb, presumably because of the inclusion of bacterial sequences, which failed the %A+T test (A+T content <50% for bacteria) but passed the similarity test (E<e^−80^ to a bacterial sequence).

### Gene Prediction (Integrated Genomics, Inc., Chicago, IL)

Genes with known homologs were initially identified utilizing BLASTX versus the ERGO non-redundant sequence database with a similarity cutoff of e^−15^. Genes identified were used to train a Hidden Markov Model-based statistical recognition program, FunGene [29] that was used to identify novel genes. Intergenic regions were further examined using Glimmer3 [30],[61] to identify missed coding sequences. ORFs were assigned identity and function using the ERGO non-redundant database and its curated annotations using procedures previously described [28].

### Accession Numbers

The *Enterocytozoon bieneusi* whole genome shotgun project has been deposited at DDBJ/EMBL/GenBank under the project accession ABGB00000000. The version described in this paper is the first version, ABGB01000000. The *E. bieneusi* genome survey dataset will also be deposited in the Biodefense and Public Health Database (BioHealthBase; http://www.biohealthbase.org).

## Supporting Information

Figure S1 Schematic of the multi-copy genes found on contigs C167 and C1951. The gene size (in nucleotides) is shown above and the start and stop coordinates are shown below. The complete list of multi-copy genes found to date is presented in Table S1.
(1.21 MB EPS)

Figure S2 Analysis of introns in *E. bieneusi*. Proteins with similarity to the *E. bieneusi* ORFs with putative introns were identified by BLAST searches and full-length sequences were aligned using ClustalW. Only the region of the alignment containing the splice and flanking anchor regions is shown. The amino acids that are present in the spliced region are boxed. The unspliced *E. bieneusi* ORF is the upper sequence (EBI_xxxxx_1) and the spliced ORF is below it (EBI_xxxxx_1a). Conserved amino acids are indicated by an aserisk, colon or dot. (A). Accession numbers for EBI_22653 alignment (elongation factor 2): NP_586452.1 (*Encephalitozoon*), Q23716 (*Cryptosporidium*, Q06193 (*Entamoeba*), XP_637051.1 (*Dictyostelium*), NP_031933.1 (*Mus*), NP_492457.1 (*Caenorhabditis*), NP_010673.1 (*Saccharomyces*), and NP_849818.1 (*Arabidopsis*). (B). Accession numbers for EBI_21989 alignment (major facilitator superfamily protein): NP_585973.1 (*Encephalitozoon*), XP_639288.1 (*Dictyostelium*), NP_013342.1 (*Saccharomyces*), NP_174489.1 (*Arabidopsis*), and NP_001055457.1 (*Oryza*). (C). Accession numbers for EBI_22717 alignment (histone-acetyltransferase-like transcription factor): NP_586258.1 (*Encephalitozoon*), AAD38202.1 (*Toxoplasma*), NP_011768.1 (*Saccharomyces*), XP_566649.1 (*Cryptococcus*), XP_62872.1 (*Cryptosporidium*), XP_639086.1 (*Dictyostelium*), NP_491173.1 (*Caenorhabditis*), NP_567002.1 (*Arabidopsis*), and NP_064389.2 (*Mus*).
(2.13 MB TIF)

Table S1 Multi-copy genes in the *E. bieneusi* genome. Seventeen multi-copy genes found in *E. bieneusi* are listed with their contig location (contig number and nucleotide position). The best *E. cuniculi* or *E. hellem* homolog match based on BLAST is shown (name, accession number, and E value). The protein assignment in the final dataset is presented if it differs from the *E. cuniculi* or *E. hellem* BLAST analysis.
(0.16 MB DOC)

Table S2 Comparison of the protein lengths of the T-complex protein (TCP) 1 subunits homologs from twelve eukaryotic organisms.
(0.11 MB DOC)

Table S3 Comparison of the protein lengths of the DNA-directed RNA polymerase III (RPC) and transcription initiation factor TFIII B subunit (IIIB) homologs from twelve eukaryotic organisms.
(0.10 MB DOC)

Table S4 Gene list of *E. bieneusi* protein-coding ORFs with assigned functions and assignment to functional categories. *E. cuniculi* proteins (gene locus tag included) within these functional categories [25] are also shown, in addition to the total number of proteins within each category for both organisms.
(1.27 MB DOC)

Table S5 Summary of the *E. bieneusi* contig data.
(0.03 MB DOC)
