# Supplementary figures and images for: Genomic Survey of the Non-Cultivatable Opportunistic Human Pathogen, Enterocytozoon bieneusi
Source: PLoS Pathog. 2009 Jan 9;5(1):e1000261. doi: 10.1371/journal.ppat.1000261 (PMC2607024; doi:10.1371/journal.ppat.1000261)

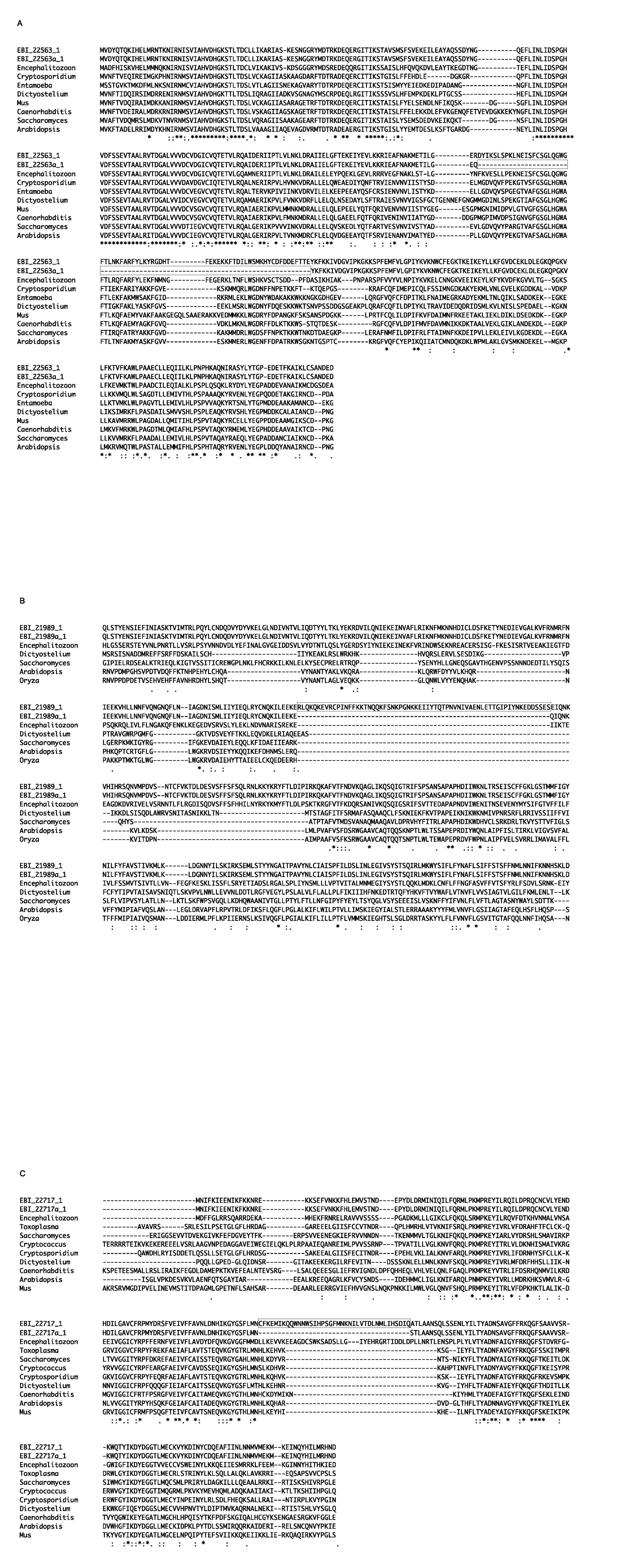

Supplement: Figure S2 — Analysis of introns in E. bieneusi. Proteins with similarity to the E. bieneusi ORFs with putative introns were identified by BLAST searches and full-length sequences were aligned using ClustalW. Only the region of the alignment containing the splice and flanking anchor regions is shown. The amino acids that are present in the spliced region are boxed. The unspliced E. bieneusi ORF is the upper sequence (EBI_xxxxx_1) and the spliced ORF is below it (EBI_xxxxx_1a). Conserved amino acids are indicated by an aserisk, colon or dot. (A). Accession numbers for EBI_22653 alignment (elongation factor 2): NP_586452.1 (Encephalitozoon), Q23716 (Cryptosporidium, Q06193 (Entamoeba), XP_637051.1 (Dictyostelium), NP_031933.1 (Mus), NP_492457.1 (Caenorhabditis), NP_010673.1 (Saccharomyces), and NP_849818.1 (Arabidopsis). (B). Accession numbers for EBI_21989 alignment (major facilitator superfamily protein): NP_585973.1 (Encephalitozoon), XP_639288.1 (Dictyostelium), NP_013342.1 (Saccharomyces), NP_174489.1 (Arabidopsis), and NP_001055457.1 (Oryza). (C). Accession numbers for EBI_22717 alignment (histone-acetyltransferase-like transcription factor): NP_586258.1 (Encephalitozoon), AAD38202.1 (Toxoplasma), NP_011768.1 (Saccharomyces), XP_566649.1 (Cryptococcus), XP_62872.1 (Cryptosporidium), XP_639086.1 (Dictyostelium), NP_491173.1 (Caenorhabditis), NP_567002.1 (Arabidopsis), and NP_064389.2 (Mus). (2.13 MB TIF) [file ppat.1000261.s002.tif]
