# Supplementary material for: Genomic Survey of the Non-Cultivatable Opportunistic Human Pathogen, Enterocytozoon bieneusi
Source: PLoS Pathog. 2009 Jan 9;5(1):e1000261. doi: 10.1371/journal.ppat.1000261 (PMC2607024; doi:10.1371/journal.ppat.1000261)
Supplement: Table S1 — Multi-copy genes in the E. bieneusi genome. Seventeen multi-copy genes found in E. bieneusi are listed with their contig location (contig number and nucleotide position). The best E. cuniculi or E. hellem homolog match based on BLAST is shown (name, accession number, and E value). The protein assignment in the final dataset is presented if it differs from the E. cuniculi or E. hellem BLAST analysis. (0.16 MB DOC) [file ppat.1000261.s003.doc]

Table S1. Multi-copy genes in the *E. bieneusi* genome. Seventeen multi-copy genes found in *E. bieneusi* are listed with their contig location (contig number and nucleotide position). The best *E. cuniculi* or *E. hellem* homolog match based on BLAST is shown (name, accession number, and E value). The protein assignment in the final dataset is presented if it differs from the *E. cuniculi* or *E. hellem* BLAST analysis.

| ***E. bieneusi* Locus Tag** | **Contig** | **Start** | **Stop** | ***E. cuniculi* (or *E. hellem*) Homolog - Top Hit** | **Accession # - Top Hit** | **E value - Top Hit** | **Protein Assignment in Final Dataset^1^** |
| --- | --- | --- | --- | --- | --- | --- | --- |
| EBI_25452 | C1044 | 22,447 | 21,689 | ER lumen protein retaining receptor (KDEL receptor 2) [*E. cuniculi* GB-M1] | CAD25518 | 4E-42 |  |
| EBI_21804 | C1044 | 23,340 | 22,618 | ER lumen protein retaining receptor (KDEL receptor 2) [*E. cuniculi* GB-M1] | CAD25518 | 8E-24 |  |
| EBI_25453 | C1044 | 24,117 | 23,404 | ER lumen protein retaining receptor (KDEL receptor 2) [*E. cuniculi* GB-M1] | CAD25518 | 2E-41 |  |
|  |  |  |  |  |  |  |  |
| EBI_22585 | C154 | 81,040 | 79,871 | Proteasome regulatory subunit YTA6 of the AAA family of ATPases *[E. cuniculi* GB-M1] | CAD25994 | 2E-28 |  |
| EBI_25889 | C154 | 82,234 | 81,086 | Proteasome regulatory subunit YTA6 of the AAA family of ATPases *[E. cuniculi* GB-M1] | CAD25994 | 2E-31 |  |
| EBI_25890 | C154 | 83,485 | 82,289 | Proteasome regulatory subunit YTA6 of the AAA family of ATPases *[E. cuniculi* GB-M1] | CAD25994 | 4E-15 |  |
| EBI_26042 | C166 | 52,524 | 51,106 | Proteasome regulatory subunit YTA6 of the AAA family of ATPases *[E. cuniculi* GB-M1] | CAD25994 | 8E-21 |  |
| EBI_22798 | C167 | 46,950 | 45,562 | Proteasome regulatory subunit YTA6 of the AAA family of ATPases *[E. cuniculi* GB-M1] | CAD25994 | 9E-31 |  |
| EBI_22799 | C167 | 48,381 | 47,038 | Proteasome regulatory subunit YTA6 of the AAA family of ATPases *[E. cuniculi* GB-M1] | CAD25994 | 6E-19 |  |
|  |  |  |  |  |  |  |  |
| EBI_22730 | C1643 | 19,135 | 20,157 | Putative 1-Acyl-sn-glycerol-3-phosphate acyltransferase *[E. cuniculi* GB-M1] | CAD25845 | 3E-28 |  |
| EBI_22731 | C1643 | 20,453 | 21,268 | Putative 1-Acyl-sn-glycerol-3-phosphate acyltransferase *[E. cuniculi* GB-M1] | CAD25845 | 8E-21 |  |
|  |  |  |  |  |  |  |  |
| EBI_26148 | C168 | 85,053 | 84,265 | Protein of the syntaxin/epimorphin family *[E.* *cuniculi* GB-M1] | CAD26066 | 1E-30 | t-SNARE complex subunit, syntaxin |
| EBI_26149 | C168 | 85,934 | 85,131 | Protein of the syntaxin/epimorphin family *[E.* *cuniculi* GB-M1] | CAD26066 | 3E-31 | t-SNARE complex subunit, syntaxin |
|  |  |  |  |  |  |  |  |
| EBI_27080 | C30 | 29,518 | 30,246 | Aquaporin-like protein [*E. hellem*] | AAQ91842 | 4E-58 | Glycerol uptake facilitator and related permeases (Major intrinsic protein family) |
| EBI_27085 | C30 | 32,586 | 33,266 | Aquaporin-like protein [*E. hellem*] | AAQ91842 | 6E-18 | Glycerol uptake facilitator related permease |
|  |  |  |  |  |  |  |  |
| EBI_21897 | C111 | 1,980 | 3,161 | Similarity to Hypothetical protein YM60_yeast *[E.* *cuniculi* GB-M1] | CAD25622 | 5E-27 | Alpha/beta hydrolase |
| EBI_21910 | C112 | 508 | 1,203 | Similarity to Hypothetical protein YM60_yeast *[E.* *cuniculi* GB-M1] | CAD25622 | 7E-31 | Alpha/beta hydrolase |
| EBI_24201 | C30 | 41,205 | 42,218 | Similarity to Hypothetical protein YM60_yeast *[E.* *cuniculi* GB-M1] | CAD25622 | 2E-33 | Alpha/beta hydrolase |
|  |  |  |  |  |  |  |  |
| EBI_23453 | C2104 | 655 | 2,271 | Glucosamine fructose-6-phosphate aminotransferase *[E.* *cuniculi* GB-M1] | CAD25661 | 1E-75 |  |
| EBI_24328 | C363 | 38,776 | 36,893 | Glucosamine fructose-6-phosphate aminotransferase *[E.* *cuniculi* GB-M1] | CAD25661 | 4E-93 |  |
| EBI_24374 | C368 | 15,588 | 17,501 | Glucosamine fructose-6-phosphate aminotransferase *[E.* *cuniculi* GB-M1] | CAD25661 | 7E-88 |  |
|  |  |  |  |  |  |  |  |
| EBI_21707 | C1012 | 82,143 | 82,640 | Peptidyl-prolyl cis-trans isomerase *[E.* *cuniculi* GB-M1] | CAD26352 | 6E-50 |  |
| EBI_22329 | C141 | 13,718 | 14,344 | Peptidyl-prolyl cis-trans isomerase *[E.* *cuniculi* GB-M1] | CAD26352 | 2E-35 |  |
|  |  |  |  |  |  |  |  |
| EBI_27186 | C367 | 9,761 | 9,144 | Protein kinase domain *[E.* *cuniculi* GB-M1] | CAD25427 | 3E-21 | Adenylate kinase related protein |
| EBI_27190 | C368 | 2,137 | 1,505 | Protein kinase domain *[E.* *cuniculi* GB-M1] | CAD25427 | 6E-21 | Adenylate kinase related protein |
|  |  |  |  |  |  |  |  |
| EBI_21796 | C1044 | 6,736 | 9,072 | Ribonucleoside diphosphate reductase *[E.* *cuniculi* GB-M1] | CAD25811 | 0 | Ribonucleoside diphosphate reductase, large chain |
| EBI_26131 | C168 | 63,236 | 60,924 | Ribonucleoside diphosphate reductase *[E.* *cuniculi* GB-M1] | CAD25811 | 0 | Ribonucleoside diphosphate reductase, large chain |
|  |  |  |  |  |  |  |  |
| EBI_25960 | C1628 | 18,940 | 20,346 | RNA-binding protein of the Pumilio family *[E.* *cuniculi* GB-M1] | CAD26083 | 1E-49 | Maternal pumilio protein |
| EBI_24304 | C363 | 7,656 | 9,185 | RNA-binding protein of the Pumilio family *[E.* *cuniculi* GB-M1] | CAD26083 | 4E-77 | Maternal pumilio protein |
|  |  |  |  |  |  |  |  |
| EBI_22580 | C154 | 73,022 | 72,075 | Ser/Thr protein phosphatase PPI-1 catalytic subunit *[E.* *cuniculi* GB-M1] | CAD25976 | 1E-122 | Serine-threonine protein phosphatase PP1 |
| EBI_25276 | C976 | 19,128 | 20,510 | Ser/Thr protein phosphatase PPI-1 catalytic subunit *[E.* *cuniculi* GB-M1] | CAD25976 | 5E-50 | Calcineurin catalytic subunit (EC 3.36) |
|  |  |  |  |  |  |  |  |
| EBI_21800 | C1044 | 10,446 | 9,106 | Serine hydroxymethyltransferase *[E.* *cuniculi* GB-M1] | CAD24889 | 8E-93 |  |
| EBI_22494 | C152 | 13,019 | 14,386 | Serine hydroxymethyltransferase *[E.* *cuniculi* GB-M1] | CAD24889 | 1E-114 |  |
|  |  |  |  |  |  |  |  |
| EBI_26075 | C167 | 26,605 | 26,147 | Similarity to 28 kDa Ribonucleoprotein (chloroplast) *[E.* *cuniculi* GB-M1] | CAD24954 | 2E-11 | Hypothetical protein |
| EBI_26413 | C1951 | 80,142 | 80,600 | Similarity to 28 kDa Ribonucleoprotein (chloroplast) *[E.* *cuniculi* GB-M1] | CAD24954 | 2E-11 | Hypothetical protein |
|  |  |  |  |  |  |  |  |
| EBI_26076 | C167 | 26,653 | 27,489 | Hypothetical protein ECU04_1510 *[E.* *cuniculi* GB-M1] | CAD25340 | 4E-23 | EMAP domain |
| EBI_23251 | C1951 | 80,094 | 79,258 | Hypothetical protein ECU04_1510 *[E.* *cuniculi* GB-M1] | CAD25340 | 4E-23 | Hypothetical protein |
|  |  |  |  |  |  |  |  |
| EBI_26095 | C167 | 57,586 | 56,546 | Similarity to Suppressor of stem loop protein 1 *[E.* *cuniculi* GB-M1] | CAD25215 | 6E-55 | Transcription/repair factor TFIIH subunit |
| EBI_22830 | C168 | 1,531 | 491 | Similarity to Suppressor of stem loop protein 1 *[E.* *cuniculi* GB-M1] | CAD25215 | 2E-54 | Transcription/repair factor TFIIH subunit |
| EBI_26368 | C1951 | 7,806 | 8,369 | Similarity to Suppressor of stem loop protein 1 *[E.* *cuniculi* GB-M1] | CAD25215 | 8E-30 | Transcription/repair factor TFIIH subunit |
|  |  |  |  |  |  |  |  |
| EBI_25411 | C1012 | 86,508 | 86,023 | Zinc finger protein *[E.* *cuniculi* GB-M1] | CAD26238 | 2E-19 | Transcriptional repressor, predicted |
| EBI_25959 | C1628 | 18,308 | 18,820 | Zinc finger protein *[E.* *cuniculi* GB-M1] | CAD26238 | 1E-18 | Transcriptional repressor, predicted |
|  |  |  |  |  |  |  |  |
| EBI_26064 | C166 | 84,252 | 80,821 | Cation-transporting ATPase *[E.* *cuniculi* GB-M1] | CAD25682 | 1E-115 |  |
| EBI_26069 | C167 | 3,640 | 242 | Cation-transporting ATPase *[E.* *cuniculi* GB-M1] | CAD25682 | 1E-117 |  |
| EBI_26421 | C1951 | 96,715 | 93,287 | Cation-transporting ATPase *[E.* *cuniculi* GB-M1] | CAD25682 | 1E-116 |  |
|  |  |  |  |  |  |  |  |
| EBI_27105 | C30 | 71,947 | 73,674 | ABC-type multidrug transport system, ATPase and permease component *[E.* *cuniculi* GB-M1] | CAD25235 | 1E-61 |  |
| EBI_22908 | C1704 | 14,319 | 12,595 | ABC-type multidrug transport system, ATPase and permease component *[E.* *cuniculi* GB-M1] | CAD25235 | 2E-65 |  |

^1^ Protein assignment in the final dataset based on best match against the GenBank NR database.
