# Supplementary material for: Genomic Survey of the Non-Cultivatable Opportunistic Human Pathogen, Enterocytozoon bieneusi
Source: PLoS Pathog. 2009 Jan 9;5(1):e1000261. doi: 10.1371/journal.ppat.1000261 (PMC2607024; doi:10.1371/journal.ppat.1000261)
Supplement: Table S2 — Comparison of the protein lengths of the T-complex protein (TCP) 1 subunits homologs from twelve eukaryotic organisms. (0.11 MB DOC) [file ppat.1000261.s004.doc]

Table S2. Comparison of the protein lengths of the T-complex protein (TCP) 1 subunits homologs from twelve eukaryotic organisms.

| **Organism** | **TCP 1α** | | | **TCP 1β** | | | **TCP 1γ** | | | **TCP 1δ** | | |  |
| --- | --- | --- | --- | --- | --- | --- | --- | --- | --- | --- | --- | --- | --- |
| *E. bieneusi* | EBI_23981 | 507^1^ | 0.0^2^ | EBI_23226 | 527 | 0.0 | EBI_24428 | 524 | 0.0 | EBI_24220 | 504 | 0.0 |  |
| *E. cuniculi* | NP_597533 | 540 | 6.5 | XP_955601 | 508 | -3.6 | NP_586139 | 519 | -4.0 | NP_584579 | 484 | -4.0 |  |
| *S. pombe* | NP_595949 | 556 | 9.7 | NP_593017 | 527 | 0.0 | NP_595810 | 528 | 4.6 | NP_595155 | 527 | 4.6 |  |
| *S. cerevisiae* | NP_010498 | 559 | 10.3 | NP_012124 | 527 | 0.0 | NP_012520 | 534 | 4.8 | NP_010138 | 528 | 4.8 |  |
| *C. neoformans* | XP_569389 | 558 | 10.1 | XP_566776 | 523 | -0.8 | XP_567673 | 567 | 6.2 | XP_571811 | 535 | 6.2 |  |
| *T. castaneum* | XP_969171 | 557 | 9.9 | XP_970646 | 534 | 1.3 | XP_973604 | 550 | 6.0 | XP_973323 | 534 | 6.0 |  |
| *D. melanogaster* | NP_524450 | 557 | 9.9 | NP_572524 | 535 | 1.5 | P48605 | 544 | 5.8 | NP_609579 | 533 | 5.8 |  |
| *M. musculus* | P11984 | 556 | 9.7 | BAA81874 | 535 | 1.5 | NP_033966 | 545 | 6.9 | NP_033967 | 539 | 6.9 |  |
| *H. sapiens* | NP_110379 | 556 | 9.7 | NP_006422 | 535 | 1.5 | CAI14167 | 545 | 6.9 | NP_006421 | 539 | 6.9 |  |
| *E. histolytica* | XP_648370 | 544 | 7.3 | XP_652436 | 533 | 1.1 | XP_650158 | 551 | 6.8 | XP_651775 | 538 | 6.8 |  |
| *C. hominis* | XP_666500 | 567 | 11.8 | XP_667713 | 531 | 0.8 | EAL35074 | 557 | 6.4 | XP_668309 | 536 | 6.4 |  |
| *A. thaliana* | AAN72063 | 545 | 7.5 | AAM61658 | 527 | 0.0 | AAO22566 | 555 | 6.4 | NP_188447 | 536 | 6.4 |  |
|  |  |  |  |  |  |  |  |  |  |  |  |  |  |
|  |  |  |  |  |  |  |  |  |  |  |  |  |  |
| **Organism** | **TCP 1ε** | | | **TCP 1η** | | | **TCP 1θ** | | | **TCP 1ζ** | | | **Average^3^** |
| *E. bieneusi* | EBI_22757 | 535 | 0.0 | EBI_22904 | 503 | 0.0 | EBI_26072 | 442 | 0.0 | EBI_27459 | 442 | 0.0 | 0.0 |
| *E. cuniculi* | NP_585855 | 532 | -0.6 | NP_586178 | 511 | 1.6 | NP_584786 | 485 | 9.7 | NP_585807 | 485 | 9.5 | 3.1 |
| *S. pombe* | NP_593277 | 546 | 2.1 | NP_596355 | 558 | 10.9 | NP_595406 | 546 | 23.5 | NP_595369 | 546 | 8.3 | 8.5 |
| *S. cerevisiae* | NP_012598 | 562 | 5.0 | NP_012424 | 550 | 9.3 | NP_012526 | 568 | 28.5 | NP_010474 | 568 | 10.5 | 10.1 |
| *C. neoformans* | XP_566476 | 548 | 2.4 | XP_566667 | 560 | 11.3 | XP_569582 | 547 | 23.8 | XP_572859 | 547 | 11.7 | 10.5 |
| *T. castaneum* | XP_968355 | 543 | 1.5 | XP_967459 | 540 | 7.4 | XP_975299 | 544 | 23.1 | XP_967748 | 544 | 7.3 | 8.6 |
| *D. melanogaster* | NP_523707 | 542 | 1.3 | NP_649835 | 544 | 8.2 | NP_610418 | 546 | 23.5 | NP_573066 | 546 | 7.9 | 8.6 |
| *M. musculus* | NP_031663 | 541 | 1.1 | CAA83274 | 544 | 8.2 | NP_033970 | 548 | 24.0 | NP_033968 | 548 | 7.5 | 8.8 |
| *H. sapiens* | NP_036205 | 541 | 1.1 | NP_006420 | 543 | 8.0 | NP_006576 | 548 | 24.0 | NP_001753 | 548 | 7.5 | 8.7 |
| *E. histolytica* | XP_655740 | 536 | 0.2 | XP_655030 | 513 | 2.0 | XP_654184 | 538 | 21.7 | XP_656288 | 538 | 9.3 | 7.5 |
| *C. hominis* | XP_668261 | 537 | 0.4 | XP_665218 | 557 | 10.7 | XP_668422 | 527 | 19.2 | XP_668555 | 527 | 7.7 | 8.9 |
| *A. thaliana* | NP_173859 | 535 | 0.0 | AAF23199 | 557 | 10.7 | AAK43867 | 549 | 24.2 | NP_197111 | 549 | 8.3 | 9.0 |

*Schizosaccharomyces pombe, S. pombe; Cryptococcus neoformans, C. neoformans; Tribolium castaneum, T. castaneum; Drosophila melanogaster, D.melanogaster; Mus musculus, M. musculus; Homo sapiens, H. sapiens; Entamoeba histolytica, E. histolytica; Cryptosporidium hominis, C. hominis; Arabidopsis thaliana, A. thaliana*.

^1^Protein length, amino acids.

^2^Difference in protein size compared to *E. bieneusi* homolog, expressed as a %.

^3^Average difference of all eight proteins, expressed as a %.
