# Supplementary material for: Genomic Survey of the Non-Cultivatable Opportunistic Human Pathogen, Enterocytozoon bieneusi
Source: PLoS Pathog. 2009 Jan 9;5(1):e1000261. doi: 10.1371/journal.ppat.1000261 (PMC2607024; doi:10.1371/journal.ppat.1000261)
Supplement: Table S3 — Comparison of the protein lengths of the DNA-directed RNA polymerase III (RPC) and transcription initiation factor TFIII B subunit (IIIB) homologs from twelve eukaryotic organisms. (0.10 MB DOC) [file ppat.1000261.s005.doc]

Table S3. Comparison of the protein lengths of the DNA-directed RNA polymerase III (RPC) and transcription initiation factor TFIII B subunit (IIIB) homologs from twelve eukaryotic organisms.

| **Organism^1^** | **RPC1** | | | **RPC2** | | | **RPC6** | | |  |
| --- | --- | --- | --- | --- | --- | --- | --- | --- | --- | --- |
| *E. bieneusi* | EBI_22530 | 1174^1^ | 0.0^2^ | EBI_26155 | 1107 | 0.0 | EBI_24426 | 240 | 0.0 |  |
| *E. cuniculi* | NP_585937 | 1349 | 14.9 | NP_586343 | 1110 | 0.3 | NP_586136 | 236 | -1.7 |  |
| *S. pombe* | NP_595506 | 1405 | 19.7 | NP_593690 | 1165 | 5.2 | NP_588398 | 301 | 25.4 |  |
| *S. cerevisiae* | NP_014759 | 1460 | 24.4 | NP_014850 | 1149 | 3.8 | NP_014400 | 317 | 32.1 |  |
| *C. neoformans* | XP_571468 | 1466 | 24.9 | XP_572718 | 1129 | 2.0 | XP_571634 | 559 | 132.9 |  |
| *T. castaneum* | XP_968165 | 1390 | 18.4 | XP_966549 | 1122 | 1.4 | XP_971496 | 301 | 25.4 |  |
| *D. melanogaster* | NP_001096987 | 1376 | 17.2 | NP_523706 | 1137 | 2.7 | NP_651029 | 293 | 22.1 |  |
| *M. musculus* | NP_001074716 | 1390 | 18.4 | NP_081699 | 1133 | 2.4 | NP_084039 | 316 | 31.7 |  |
| *H. sapiens* | NP_008986 | 1390 | 18.4 | NP_060552 | 1133 | 2.4 | NP_006457 | 316 | 31.7 |  |
| *E. histolytica* | XP_653748 | 1379 | 17.5 | XP_654890 | 1122 | 1.4 | NF |  |  |  |
| *C. hominis* | XP_667260 | 1869 | 59.2 | NF^3^ |  |  | XP_668110 | 319 | 32.9 |  |
| *A. thaliana* | NP_200812 | 1376 | 17.2 | NP_199327 | 1161 | 4.9 | NP_197760 | 230 | -4.2 |  |
|  |  |  |  |  |  |  |  |  |  |  |
|  |  |  |  |  |  |  |  |  |  |  |
| **Organism** | **RPC10** | | | **IIIB** | | | **RPC19** | | | **Average^4^** |
| *E. bieneusi* | EBI_26246 | 95 | 0.0 | EBI_22687 | 391 | 0.0 | EBI_27693 | 107 | 0.0 | 0.0 |
| *E. cuniculi* | NP_597560 | 104 | 9.5 | NP597392 | 395 | 1.0 | NP_586365 | 104 | -2.8 | 3.5 |
| *S. pombe* | NP_593235 | 109 | 14.7 | NP_596265 | 500 | 27.9 | NP_593118 | 125 | 16.8 | 18.3 |
| *S. cerevisiae* | NP_010330 | 110 | 15.8 | NP_011762 | 596 | 52.4 | NP_014286 | 142 | 32.7 | 26.9 |
| *C. neoformans* | XP_571795 | 132 | 39.0 | XP_571484 | 691 | 76.7 | XP_568809 | 155 | 44.9 | 53.4 |
| *T. castaneum* | XP_972311 | 108 | 13.7 | XP_973702 | 617 | 57.8 | XP_975661 | 105 | -1.8 | 19.1 |
| *D. melanogaster* | NP_001027443 | 108 | 13.7 | AAF72066 | 662 | 69.3 | Q9VIZ0 | 105 | -1.8 | 20.5 |
| *M. musculus* | NP_080177 | 108 | 13.7 | NP_082469 | 676 | 72.9 | P97304 | 133 | 24.3 | 27.2 |
| *H. sapiens* | Q9Y2Y1 | 108 | 13.7 | NP_001510 | 677 | 73.1 | NP_057056 | 133 | 24.3 | 27.3 |
| *E. histolytica* | XP_654238 | 107 | 12.6 | XP_650799 | 546 | 39.6 | NF |  |  | 17.8 |
| *C. hominis* | NF |  |  | XP_667830 | 646 | 65.2 | NF |  |  | 52.4 |
| *A. thaliana* | NP_171629 | 106 | 11.6 | AAD32827 | 565 | 44.5 | NP_180514 | 122 | 14.0 | 14.7 |

*Schizosaccharomyces pombe, S. pombe; Cryptococcus neoformans, C. neoformans; Tribolium castaneum, T. castaneum; Drosophila melanogaster, D.melanogaster; Mus musculus, M. musculus; Homo sapiens, H. sapiens; Entamoeba histolytica, E. histolytica; Cryptosporidium hominis, C. hominis; Arabidopsis thaliana, A. thaliana*.

^1^Protein length, amino acids.

^2^Difference in protein size compared to *E. bieneusi* homolog, expressed as a %.

^3^NF, not present in organism.

^4^Average difference of all six proteins, expressed as a %. Exceptions are *E. histolytica and C. hominis*.
