# Supplementary material for: Genomic Survey of the Non-Cultivatable Opportunistic Human Pathogen, Enterocytozoon bieneusi
Source: PLoS Pathog. 2009 Jan 9;5(1):e1000261. doi: 10.1371/journal.ppat.1000261 (PMC2607024; doi:10.1371/journal.ppat.1000261)
Supplement: Table S4 — Gene list of E. bieneusi protein-coding ORFs with assigned functions and assignment to functional categories. E. cuniculi proteins (gene locus tag included) within these functional categories [25] are also shown, in addition to the total number of proteins within each category for both organisms. (1.27 MB DOC) [file ppat.1000261.s006.doc]

Table S4. Gene list of *E. bieneusi* protein-coding ORFs with assigned functions and assignment to functional categories. *E. cuniculi* proteins (gene locus tag included) within these functional categories [22] are also shown, in addition to the total number of proteins within each category for both organisms.

| ***E. bieneusi* Homolog (EBI Locus Tag)** | ***E. cuniculi* Proteins Within Functional Categories [22]** | ***E. cuniculi* Gene Locus Tag [22]** | **Total # *E. cuniculi* Proteins** | **Total # *E. bieneusi* Proteins** |
| --- | --- | --- | --- | --- |
|  |  |  |  |  |
|  | **I. METABOLISM** |  | **79** | **44-45** |
|  |  |  |  |  |
|  | ***Amino-acid metabolism*** |  | **4** | **3** |
|  | asparagine Sase (ASNH METJA) | ECU04_0840 |  |  |
| 24650, 27328 | deoxyhypusine Sase (DHYS SCHPO) | ECU09_0910 |  |  |
| 22553 | diphthine Sase (DPH5 yeast) | ECU11_1430 |  |  |
| 26033 | ornithine decarboxylase, w. sim (DCOR yeast) | ECU08_0360 |  |  |
|  |  |  |  |  |
|  | ***Nitrogen and sulfur metabolism*** |  | **3** | **2** |
|  | NGG1-interacting factor 3 (NIF3 yeast) | ECU07_0100 |  |  |
| 23264 | NIFS-like prt (NFS1 CANAL) | ECU11_1770 |  |  |
| 27682 | NIFU-like prt (NIFU RICPR) | ECU01_0510 |  |  |
|  |  |  |  |  |
|  | ***Nucleotide metabolism*** |  |  |  |
|  |  |  |  |  |
|  | ***Purine-ribonucleotide metabolism*** |  | ***6*** | **3** |
|  | dihydrofolate RDase (DYR ENCCU)^2^ | ECU01_0170 |  |  |
|  | dihydrofolate RDase (DYR ENCCU)^2^ | ECU01_1450 |  |  |
|  | dihydrofolate RDase (DYR ENCCU)^2^ | ECU08_0080 |  |  |
| 26696 | guanylate kinase (KGUA yeast) | ECU01_1220 |  |  |
| 21800, 22494^1a^ | serine hydroxymethylTase (GLYC yeast)^2^ | ECU01_0190 |  |  |
| 21800, 22494^1a^ | serine hydroxymethylTase (GLYC yeast)^2^ | ECU01_1420 |  |  |
|  |  |  |  |  |
|  | ***Pyrimidine-ribonucleotide metabolism*** |  | **4** | **2** |
|  | CTP Sase (PYRG MOUSE)^2^ | ECU11_0880 |  |  |
|  | CTP Sase (PYRG MOUSE)^2^ | ECU11_0480 |  |  |
| 26854 | cytidylate kinase (KCY BACSU) | ECU03_1270 |  |  |
| 27097 | thymidylate kinase (KTHY yeast) | ECU04_1220 |  |  |
|  |  |  |  |  |
|  | ***Deoxyribonucleotide metabolism*** |  | **11** | **10-11** |
| 25403 | cytidine and dCMPdeaminase (TAD2 yeast) | ECU09_1960 |  |  |
| 25911, 26829, 26951, 27561 | deoxyuridine 5'-triP nucleotidohydrolase (DUT CHVP1) | ECU05_0280 |  |  |
| 24637 | deoxyuridine 5'-triP nucleotidohydrolase (DUT yeast) | ECU06_0430 |  |  |
| 21807^1b^, 26391 | glutaredoxin (GLRX ORYZA) | ECU08_1380 |  |  |
| 21807^1b^ | glutaredoxin (GLR2 SCHPO) | ECU09_1375 |  |  |
| 21796, 26131 | ribonucleoside-diP RDase, large sub (RIR1 CAEEL) | ECU10_0920 |  |  |
| 21834, 22039, 22745, 22942, 22943, 23034, 23134, 23293, 23526, 23669, 23893, 23907, 23921, 24269, 24681, 24974, 25250, 26336, 26359, 26721, 26726, 26912, 26928, 27341 | ribonucleoside-diP RDase, small sub (RIR2 BRARE) | ECU06_0730 |  |  |
| 24340, 27158 | thymidine kinase (KITH ENCCU) | ECU01_0740 |  |  |
| 22497^1a^ | thymidylate Sase (TYSY ENCCU)^2^ | ECU01_0180 |  |  |
| 22497^1a^ | thymidylate Sase (TYSY ENCCU)^2^ | ECU01_1430 |  |  |
| 22497^1a^ | thymidylate Sase (TYSY ENCCU)^2^ | ECU08_0090 |  |  |
|  |  |  |  |  |
|  | ***Polynucleotide degradation*** |  | **2** | **2** |
| 25826 | deoxyribonuclease, TATD fam (TATD ECOLI) | ECU03_0200 |  |  |
| 23230 | ribonuclease, HII fam (YNH2 yeast) | ECU03_0670 |  |  |
|  |  |  |  |  |
|  | ***Other*** |  | **6** | **2** |
| 25977 | ATP-Bprt, MRP/MBP35 fam (NB35 yeast) | ECU08_0740 |  |  |
|  | bis (5'-adenosyl) triphosphatase, HIT fam (FHIT HUMAN) | ECU10_0480 |  |  |
|  | bis (5'nucleosyl) tetraphosphatase, HIT fam (APH1 SCHPO) | ECU03_0880 |  |  |
| 24673 | nucleoside diP kinase A (NDKA FLABI) | ECU06_1530 |  |  |
|  | nucleoside triphosphatase, HAM1 fam (HAM1 yeast) | ECU04_1180 |  |  |
|  | nucleoside triphosphatase, HAM1 fam (NTPA METJA) | ECU05_0870 |  |  |
|  |  |  |  |  |
|  | ***Phosphate metabolism*** |  | **5** | **5** |
| 25558 | acid phosphatase (PPAY CAEEL) | ECU07_0180 |  |  |
| 27246 | inorganic pyrophosphatase (IPYR DROME) | ECU10_0340 |  |  |
| 22559 | inositol polyphosphate-5 phosphatase, put (INP54 yeast) | ECU10_1370 |  |  |
| 21719 | involved in polyphosphate metabolism (PHM8 yeast) | ECU05_1170 |  |  |
| 23261 | polyphosphate synthase, put (VTC4 yeast) | ECU06_1420i |  |  |
|  |  |  |  |  |
|  | ***C-compound and carbohydrate metabolism*** |  | **9** | **7** |
| 24204 | α-1,2-mannosyl Tase, KTR fam (YUR1 yeast) | ECU04_1130 |  |  |
|  | acetyl-CoA Sase (ACS1 yeast) | ECU05_0310 |  |  |
| 21817 | acidic endochitinase (CHIP BETVU) | ECU09_1320 |  |  |
|  | aldose RDase (ALDR MOUSE) | ECU01_0970 |  |  |
| 22038, 22115, 21677, 22308, 22322, 22374, 22461, 22481, 22617, 22954, 23212, 23517, 23598, 23606, 23833, 24073, 24081, 24083, 24169, 24127, 24140, 24152, 24679, 24725, 24825, 25019, 25144, 25627, 25653, 25711, 25935, 26211, 26360, 26906, 26942, 27057, 27351 | chitin synthase 1 (CHS1 CRYNE) | ECU01_1390 |  |  |
| 26397 | glucosamine-P N-acetylTase (GNA1 CAEEL) | ECU07_1760 |  |  |
| 23453, 24328, 24374 | glucosamine-fructose-6-P aminoTase (GFA2 HUMAN) | ECU07_1280 |  |  |
| 21809 | phosphoacetylglucosamine mutase (PCM1 SCHPO) | ECU01_0650 |  |  |
| 27147 | UDP-N-acetylglucosamine pyrophosphorylase (UAP1 yeast) | ECU11_1780 |  |  |
|  |  |  |  |  |
|  | ***Lipid, fatty acid and isoprenoid metabolism*** |  |  |  |
|  |  |  |  |  |
|  | ***Biosynthesis*** |  | **21** | **5** |
| 24437 | 1-acyl-sn-glycerol-3-P acylTase (PLSC yeast) | ECU10_0310 |  |  |
| 26449 | 1-acyl-sn-glycerol-3-P acylTase, put (Y205 HUMAN) | ECU04_1280 |  |  |
| 22730, 22731 | 1-acyl-sn-glycerol-3-P acylTase, put (YBP2 yeast) | ECU10_1260 |  |  |
|  | 1,2-DAG ethanolamine phosphoTase (EPT1 yeast) | ECU11_0620 |  |  |
|  | 3-hydroxy-3-methylglutaryl-CoA RDase (HMD1 DICDI) | ECU10_1720 |  |  |
|  | 3-hydroxy-3-methylglutaryl-CoA Sase (HMC2 BLAGE) | ECU10_0510 |  |  |
|  | CDP-DAG inositol 3-phosphatidylTase (PIS HUMAN) | ECU01_1000 |  |  |
|  | CDP-DAG serine O-phosphatidylTase (PEL1 yeast) | ECU11_0850 |  |  |
| 23227 | CDP-DAG synthase (CDS1 yeast) | ECU05_1250 |  |  |
|  | choline-P cytidylylTase (CPCT MOUSE) | ECU10_1590 |  |  |
|  | farnesyl-PP Sase (FPPS HELAN) | ECU11_1810 |  |  |
|  | isopentenyl-diP δ-isomerase II (IDI2 ARATH) | ECU02_0230 |  |  |
| 26133^1b^ | long-chain-fatty-acid-CoA ligase (LCFX RAT) | ECU10_0890 |  |  |
| 26133^1b^ | long-chain-fatty-acid-CoA ligase (LCFX RAT) | ECU10_0910 |  |  |
|  | mevalonate kinase (KIME yeast) | ECU09_1780 |  |  |
|  | mevalonate kinase, w.sim. (KIME PYRHO) | ECU10_1510 |  |  |
|  | mevalonate-PP decarboxylase (ER19 yeast) | ECU06_0490 |  |  |
|  | N-acetylglucosaminyl-phosphatidylinositol biosynthetic protein (GPI3 yeast) | ECU09_1210 |  |  |
|  | serine palmitoyl Tase, sub 1 (LCB1 HUMAN) | ECU02_0860 |  |  |
|  | serine palmitoyl Tase, sub 2 (LCB2 HUMAN) | ECU05_0730 |  |  |
|  | sterol ester Tase (AREH SCHPO) | ECU10_0300 |  |  |
|  |  |  |  |  |
|  | ***Degradation*** |  | **5** | **2** |
|  | acetyl-CoA-C-acyl Tase (ATOB HAEIN) | ECU06_0940 |  |  |
|  | acyl-CoA thioesterase II (TESB ECOLI) | ECU05_1520 |  |  |
| 27477 | glycerophosphoryldiester phosphodiesterase, w.sim (GLPQ BACSU) | ECU01_1380 |  |  |
| 26380 | lipase, put (CVT17 yeast) | ECU04_0660 |  |  |
|  | phospholipase D2 (PLD2 RAT) | ECU11_1180 |  |  |
|  |  |  |  |  |
|  | ***Other*** |  | **3** | **1** |
| 25212 | oxidoRDase, short chain DHase/RDase fam, put (YKF5 yeast) | ECU08_1150 |  |  |
|  | oxidoRDase, short-chain DHase/RDase fam, put (DLTE BACSU) | ECU10_0560 |  |  |
|  | oxidoRDase, short-chain DHase/RDase fam, put (YAEB SCHPO) | ECU11_1070 |  |  |
|  |  |  |  |  |
|  | **II. ENERGY** |  | **23** | **3** |
|  |  |  |  |  |
|  | ***Glycolysis*** |  | **14** | **2** |
|  | 6-phosphofructokinase A (K6PF CANFA) | ECU03_0680 |  |  |
|  | enolase (ENO FASHE) | ECU10_1690 |  |  |
|  | fructose-*bis*P aldolase (ALFB HUMAN) | ECU01_0240 |  |  |
|  | glucose-6-P isomerase (G6PI PIG) | ECU05_0650 |  |  |
| 24602 | glyceraldehyde-3-P DHase (G3P2 BACSU) | ECU07_0800 |  |  |
| 22068, 22101, 23369, 23639, 23923, 24094, 24548, 25625, 25641, 25668, 25722, 25756, 26195, 26210, 26213, 26569, 26788, 26838, 26989, 27379, 27627 | glycerol-3-P DHase A (GPDA FUGRU) | ECU05_0270 |  |  |
|  | glycerol-3-P DHase, mito. (GPDM CAEEL) | ECU10_0870 |  |  |
|  | hexokinase (HXK SCHMA) | ECU11_1540 |  |  |
|  | phosphoglycerate kinase (PGK KLULA) | ECU05_0320 |  |  |
|  | phosphoglycerate mutase (PGMI ANTSP) | ECU10_1060 |  |  |
|  | pyruvate DHase E1, α sub (ODPA SCHPO) | ECU09_1040 |  |  |
|  | pyruvate DHase E1, β sub (ODPB RICPR) | ECU04_1160 |  |  |
|  | pyruvate kinase (KPYK YARLI) | ECU09_0640 |  |  |
|  | triose phosphate isomerase (TPIS SCHJA) | ECU11_0230 |  |  |
|  |  |  |  |  |
|  | ***Pentose phosphate pathway*** |  | **5** | **1** |
|  | 6-phosphogluconate DHase (6PGD SALTY) | ECU05_0860 |  |  |
|  | D-ribose-5-P isomerase (RPIA METTH) | ECU10_0180 |  |  |
|  | D-ribulose-5-P 3-epimerase (RPE METJA) | ECU06_1040 |  |  |
| 25750 | glucose-6-P 1-DHase (G6PD yeast) | ECU08_1850 |  |  |
|  | transketolase 1 (TKT1 yeast) | ECU06_0120 |  |  |
|  |  |  |  |  |
|  | ***trehalose metabolism*** |  | **4** | **0** |
|  | α,α-trehalase (TREA BOMMO) | ECU02_1370 |  |  |
|  | α,α-trehalose-P Sase, cat sub (TPSA ASPNG) | ECU01_0800 |  |  |
|  | trehalose-6-P phosphatase (TPS2 yeast) | ECU01_0870 |  |  |
|  | UTP-glucose-1-P uridylTase (UDPG yeast) | ECU03_0280 |  |  |
|  |  |  |  |  |
|  | **III. CELL GROWTH, CELL DIVISION AND DNA SYNTHESIS** |  | **136** | **108-114** |
|  |  |  |  |  |
|  | ***Cell growth and cell polarity*** |  | **11** | **9** |
| 21881, 22743, 24412, 24758, 24759, 24773, 26184, 26883, 27317, 27417, 27612 | calcineurin, β sub (CALB NAEGR) | ECU08_0160 |  |  |
| 24360, 26046, 27349 | calcium-Bprt, recoverin subfam (NCS1 yeast) | ECU04_0810 |  |  |
| 21798^1b^ | GTP-Bprt, GTP1/OBG fam (DRG1 XENLA) | ECU05_0410 |  |  |
| 21798^1b^ | GTP-Bprt, GTP1/OBG fam (DRG2 HUMAN) | ECU08_1270 |  |  |
| 24856 | GTP-Bprt, GTP1/OBG fam (FEOB METJA) | ECU05_080 |  |  |
| 21691 | involved in plasmid maintenance, respiration and cell proliferation (SMP2 yeast) | ECU02_0200 |  |  |
| 27245 | RAS-related GTP-Bprt (RAC1 CAEEL) | ECU03_1560 |  |  |
| 21713 | ser/thr protein kinase (KIN1 SCHPO) | ECU03_0980 |  |  |
| 24439 | required for normal growth,SUA5/YRDC/YC10/YWLC fam (YWLC BACSU) | ECU09_1610 |  |  |
| 27688 | suppressor of temperature-sensitive growth of yeast MRP1 mutants (SOH1 yeast) | ECU11_0360 |  |  |
|  | suppressor of BEM1/BUD5 (BE46 SCHPO) | ECU09_0730 |  |  |
|  |  |  |  |  |
|  | ***Meiosis*** |  | **2** | **0** |
|  | meiotic recombination prt (RE12 SCHPO) | ECU04_1110 |  |  |
|  | MEI2-related prt (MES1 SCHPO) | ECU06_0740 |  |  |
|  |  |  |  |  |
|  | ***DNA synthesis and replication*** |  |  |  |
|  |  |  |  |  |
|  | ***DNA directed DNA polymerases*** |  | **8** | **8** |
| 23248, 23858, 24588 | DNA pol α, sub A (DPOA SCHPO) | ECU05_0990 |  |  |
| 27250 | DNA pol α, sub B (DPO2 rat) | ECU10_1080 |  |  |
| 26384 | DNA pol/primase, large sub (PRI2 HUMAN) | ECU05_1210 |  |  |
| 25549 | DNA pol/primase, small sub (PRI1 MOUSE) | ECU08_0630 |  |  |
| 23248 | DNA pol δ, large sub (DPOD CANAL) | ECU09_0430 |  |  |
| 24597 | DNA pol δ, small sub (DPD2 MOUSE) | ECU09_0770 |  |  |
| 27447 | DNA pol δ, PCNA (PCNA BRANR) | ECU05_1030 |  |  |
| 26115 | DNA pol ε, cat sub (DPOE SCHPO) | ECU10_1210 |  |  |
|  |  |  |  |  |
|  | ***DNA replication factors A and C*** |  | **8** | **8** |
| 22900 | DNA rep factor A, prt 1 (RFA1 SCHPO) | ECU10_0600 |  |  |
| 24358 | DNA rep factor A, prt 2 (RFA2 HUMAN) | ECU06_0360 |  |  |
| 26441 | DNA rep factor A, prt 3, w.sim (RFA3 SCHPO) | ECU07_0950 |  |  |
| 24332 | DNA rep factor C sub (RFC1 yeast) | ECU05_1530 |  |  |
| 27210 | DNA rep factor C sub (RFC2 yeast) | ECU02_0680 |  |  |
| 25388 | DNA rep factor C sub (RFC3 yeast) | ECU02_0290 |  |  |
| 22840 | DNA rep factor C sub (AC13 HUMAN) | ECU10_0780 |  |  |
| 21816 | DNA rep factor C sub (RFC5 yeast) | ECU09_1330 |  |  |
|  |  |  |  |  |
|  | ***DNA replication licensing factors, MCM family*** |  | **8** | **8** |
| 22681 | MCM2 (CC19 SCHPO) | ECU04_0850 |  |  |
| 22554 | MCM3 (MCM3 SCHPO) | ECU08_0290 |  |  |
| 22567 | MCM4 (MCM4 SCHPO) | ECU02_1150 |  |  |
| 22535 | MCM4 (MCM4 DROME) | ECU11_0800 |  |  |
| 24357 | MCM5 (MCM5 yeast) | ECU06_0340 |  |  |
| 24873 | MCM6 (MCM6 MOUSE) | ECU05_0780 |  |  |
| 25571 | MCM7 (MCM7 MOUSE) | ECU07_0490 |  |  |
| 21805 | MCM7 (MCM7 XENLA) | ECU09_1360 |  |  |
|  |  |  |  |  |
|  | ***DNA topoisomerases*** |  | **3** | **3** |
| 25447 | DNA topoisomerase I (TRF5 yeast) | ECU06_1520 |  |  |
| 23976 | DNA topoisomerase II (TOP2 SCHPO) | ECU04_0350 |  |  |
| 23235 | DNA topoisomerase III (TOP3 SCHPO) | ECU04_1070 |  |  |
|  |  |  |  |  |
|  | ***Other*** |  | **7** | **7** |
| 21974 | ATP-dep DNA-binding helicase, RAD3/XPD fam (CHL1 yeast) | ECU02_1090 |  |  |
| 23273 | ATP-dep DNA-binding helicase, RAD3/XPD fam (CHL1 yeast) | ECU08_1120 |  |  |
| 23275 | DNA helicase (DNA2 yeast) | ECU09_1800 |  |  |
| 23241 | DNA ligase (DNL1 MOUSE) | ECU02_1220 |  |  |
| 25417 | origin recognition complex, sub 1 (ORC1 DROME) | ECU03_1030 |  |  |
| 22816 | origin recognition complex, sub 2 (ORC2 ARATH) | ECU10_1130 |  |  |
| 24665 | telomerase, cat sub (TERT SCHPO) | ECU09_0310 |  |  |
|  |  |  |  |  |
|  | ***Recombination and DNA repair*** |  | **22** | **18-19** |
| 22771, 24228, 24800, 26923 | ATP-dep DNA helicase (HUS2 SCHPO) | ECU07_1130 |  |  |
| 21727^1b^ | casein kinase I (HHP2 SCHPO) | ECU03_0910 |  |  |
| 21727^1b^, 24640 | casein kinase I (KC1A BOVINE) | ECU11_1980 |  |  |
| 22325 | chromodomain DNA-Bprt, SNF2/RAD54 fam (CHD2 HUMAN) | ECU01_0350 |  |  |
| 24371 | DNA mismatch repair prt, MUTL/HEXB fam (MLH1 HUMAN) | ECU05_0300 |  |  |
| 22874 | DNA mismatch repair prt, MUTL/HEXB fam (HEXB STRPN) | ECU11_1260 |  |  |
| 24871 | DNA mismatch repair prt, MUTS fam (MSH2 HUMAN) | ECU03_0540 |  |  |
| 26134 | DNA mismatch repair prt, MUTS fam (MSH6 yeast) | ECU10_0710 |  |  |
| 22725 | DNA repair helicase, RAD3/XPD subfam (RAD3 yeast) | ECU06_0200 |  |  |
| 23223 | DNA repair helicase RAD25 (RA25 yeast) | ECU01_1060 |  |  |
|  | DNA repair prt, RAD4/XPC fam (XPC MOUSE) | ECU01_0450 |  |  |
|  | DNA repair prt, RAD14/XPA fam (XPA XENLA) | ECU10_1170 |  |  |
| 24194 | DNA repair prt RAD50 (RA50 yeast) | ECU07_0610 |  |  |
|  | DNA repair prt, XPF/ERCC4/RAD1 fam (MEI9 DROME) | ECU08_0760 |  |  |
| 23231 | double-strand break DNA repair prt, MRE11/RAD32 fam (RA32 SCHPO) | ECU05_1280 |  |  |
| 22917 | exonuclease 1 (EXO1 SCHPO) | ECU11_0760 |  |  |
| 21962 | exonuclease, put (YSM4 CAEEL) | ECU07_0380 |  |  |
| 21987 | recombination and DNA repair prt RAD18 (RA18 SCHPO) | ECU07_0390 |  |  |
| 22325, 23268, 24339 | recombination and DNA repair prt RAD26 (RA26 yeast) | ECU09_0410 |  |  |
| 24364 | recombination and DNA repair prt SNF2/RAD54 fam (YA83 SCHPO) | ECU06_0820 |  |  |
| 24334, 26162 | RUVB-like DNA helicase (RUVB MYCLE) | ECU09_1390 |  |  |
| 24424 | uracyl-DNA glycosylase (UNG HUMAN) | ECU10_0530 |  |  |
|  |  |  |  |  |
|  | ***Cell cycle control and mitosis*** |  |  |  |
|  |  |  |  |  |
|  | ***Protein kinases*** |  | **19** | **12-17** |
| 22100, 22373, 22470, 23135, 23314, 23428, 25009, 25026, 25161, 25525, 25533, 25624, 25721, 26209, 26214, 26584, 27048 | casein kinase II, α sub (KC2A CAEEL) | ECU05_1510 |  |  |
| 22839 | casein kinase II, β sub KC2B DROME) | ECU10_1200 |  |  |
| 22516 | CDC 68 (Start, general transcription) (CC68 yeast) | ECU03_0420 |  |  |
| 22495^1b^, 24337^1b^, 24432^1b^, 24644^1b^, 24928^1b^ | cell cycle protein kinase CDC2 (CC2A ANTMA) | ECU11_0960 |  |  |
| 24432^1b^ | cell cycle protein kinase CDK2 (CDK2 CARAU) | ECU08_1920 |  |  |
| 22495^1b^ | cell cycle protein kinase, CDC2/CDKX subfam (CC22 MEDSA) | ECU08_0230 |  |  |
| 22495^1b^, 24337^1b^, 24432^1b^, 24644^1b^ | cell cycle protein kinase, CDC2/CDKX subfam (CDKA HUMAN) | ECU11_1290 |  |  |
| 22495^1b^, 24332^1b^, 243371b, 24644^1b^, 24928^1b^ | cell cycle protein kinase, CDC2/CDKX subfam (CDK9 CAEEL) | ECU08_1790 |  |  |
| 24444^1a^ | cell cycle protein kinase, CDC7 subfam (HSK1 SCHPO)^2^ | ECU08_1960 |  |  |
| 24444^1a^ | cell cycle protein kinase, CDC7 subfam (HSK1 SCHPO)^2^ | ECU10_0120 |  |  |
| 24437, 24644^1b^, 26558 | cyclin-dep ser/thr protein kinase (KI28 yeast) | ECU02_1450 |  |  |
| 26123 | mitogen-activated protein kinase (MK16 yeast) | ECU10_0830 |  |  |
| 21713^1b^, 25561^1b^ | ser/thr protein kinase (CHK1 SCHPO) | ECU03_0620 |  |  |
| 21713^1b^, 22495^1b^, 24337^1b^, 24432^1b^, 24644^1b^ | ser/thr protein kinase (MRK1 yeast) | ECU07_1270 |  |  |
| 27532 | ser/thr protein kinase (PKPA PHYB) | ECU05_0630 |  |  |
| 24207 | ser/thr protein kinase (SPK1 yeast) | ECU02_0550 |  |  |
| 24337^1b^, 24644^1b^ | ser/thr protein kinase (WEE1 XENLA) | ECU08_1620 |  |  |
| 25561^1b^ | ser/thr/tyr protein kinase (IPL1 yeast) | ECU07_0360 |  |  |
| 22495^1b^ | thr/tyr protein kinase (MPS1 yeast) | ECU02_0510 |  |  |
|  |  |  |  |  |
|  | ***Protein phosphatases*** |  | **10** | **6** |
| 23242, 26094 | protein tyrosine phosphatase (PTPE MOUSE) | ECU04_0300 |  |  |
| 23222 | ser/thr protein phosphatase 1-1, cat sub (PP11 yeast) | ECU09_0980 |  |  |
| 22580, 22587 | ser/thr protein phosphatase 1-1, cat sub (PP11 CAEEL) | ECU11_0660 |  |  |
| 22876^1b^ | ser/thr protein phosphatase 1-1, γ cat sub (PPP5 HUMAN) | ECU05_0440 |  |  |
| 22876^1b^ | ser/thr protein phosphatase 2-A, α sub (P2AA CHICK) | ECU04_0700 |  |  |
|  | ser/thr protein phosphatase 2-A, β sub (2A5G HUMAN) | ECU04_0200 |  |  |
|  | ser/thr protein phosphatase 2-A, reg sub A (2AAA DROME) | ECU09_1490 |  |  |
| 27457 | ser/thr protein phosphatase 2-A, reg sub B (2ABD RAT) | ECU05_0810 |  |  |
|  | ser/thr protein phosphatase 2-C (P2C1 ARATH) | ECU07_1520 |  |  |
| 23811, 23986 | PP2A activator (PTPA) | ECU06_0720 |  |  |
|  |  |  |  |  |
|  | ***Cyclins*** |  | **4** | **2** |
| 24872 | cyclin B, G2/M specific (CG2B MEDSA) | ECU08_0260 |  |  |
|  | cyclin B-like prt (CG2B CARAU) | ECU04_1460 |  |  |
| 22735 | cyclin C-like prt, G1/S transition (CG1C ORYZA) | ECU09_0840 |  |  |
|  | cyclin C-like (or K) prt (CYCK HUMAN) | ECU01_1150 |  |  |
|  |  |  |  |  |
|  | ***Chromosome segregation proteins, SMC family*** |  | **5** | **5** |
| 24197 | chromosome segregation prt (CUT3 SCHPO) | ECU07_0680 |  |  |
| 27481 | chromosome segregation prt (CUT4 SCHPO) | ECU01_1160 |  |  |
| 25401 | chromosome segregation prt (P115 MYCPN) | ECU09_1910 |  |  |
| 24210 | chromosome segregation prt (SMC1 yeast) | ECU04_0930 |  |  |
| 25402 | chromosome segregation prt, put (YDZ2 SCHPO) | ECU11_2000 |  |  |
|  | ***Other*** |  | **16** | **13** |
| 23125 | anaphase promoting complex sub (CDC23 yeast) | ECU06_1430 |  |  |
| 24652 | anaphase promoting complex sub (CUT9 SCHPO) | ECU06_0150 |  |  |
| 25018, 26508, 26535, 26660, 26764, 26766, 26995, 27110 | CDC50-like prt, put (YCY4 yeast) | ECU06_1450 |  |  |
| 24857 | centromere/microtubule-Bprt (CBF5 SCHPO) | ECU05_1020 |  |  |
| 27088 | cullin (CUL2 CAEEL) | ECU07_0860 |  |  |
| 26015 | cullin (CUL6 CAEEL) | ECU06_0880 |  |  |
| 21695^1a^ | interacts with B-type cyclin, NAP/SET fam (SET DROME)^2^ | ECU01_0210 |  |  |
| 21695^1a^ | interacts with B-type cyclin, NAP/SET fam (SET DROME)^2^ | ECU01_1400 |  |  |
| 24662, 24674 | RAS suppressor (RSU1 HUMAN) | ECU11_0170 |  |  |
| 23979 | required for microtubule-dep processes prior to anaphase (CC20 yeast) | ECU04_1250 |  |  |
| 26454 | required for spindle pole body duplication (CUT1 SCHPO) | ECU07_0350 |  |  |
|  | required for mitosis (D1B1 yeast) | ECU02_1270 |  |  |
|  | required for mitosis and meiosis (PELO DROME) | ECU03_1380 |  |  |
|  | similarity to CDC4 (CC4 yeast) | ECU01_1300 |  |  |
| 25684 | similarity to CDC20, put (YGA3 yeast) | ECU03_1520 |  |  |
| 22230, 23771, 25942, 26663, 26759 | spindle pole body prt (SAD1 SCHPO) | ECU11_1590 |  |  |
|  |  |  |  |  |
|  | ***Cytokinesis*** |  | **9** | **7-8** |
| 25857 | periodic tryptophan prt 1 (PWP1 HUMAN) | ECU03_1230 |  |  |
|  | periodic tryptophan prt 2 (PWP2 yeast) | ECU05_0190 |  |  |
| 24308 | septin (C10H MOUSE) | ECU11_1950 |  |  |
| 24591 | septin (CC10 yeast) | ECU09_0820 |  |  |
| 26394 | septin-like prt (SPN4 SCHPO) | ECU01_1370 |  |  |
| 22789 | ser/thr protein kinase required for actin ring and septation (PLO1 SCHPO) | ECU01_0630 |  |  |
| 25572^1b^ | similarity to SKT5 (interacts with septins), put (YAIA SCHPO) | ECU11_0430 |  |  |
| 21992 | similarity to SKT5 (interacts with septins), put (YBEQ ECOLI) | ECU02_1030 |  |  |
| 25572^1b^, 27424 | similarity to SKT5 (interacts with septins), put (YBEQ ECOLI) | ECU06_0540 |  |  |
|  |  |  |  |  |
|  | ***Other*** |  | **4** | **3** |
| 25884 | DNA hexamer-Bprt (HEXP LEIMA) | ECU11_0650 |  |  |
| 24925, 24931 | N6-adenine-specific DNA methylase (N6M1 HUMAN) | ECU06_0610 |  |  |
|  | N6-adenine methylTase, put (Y284 METJA) | ECU08_1860 |  |  |
| 23129 | nucleotide-Bprt (NB35 yeast) | ECU11_1190 |  |  |
|  |  |  |  |  |
|  | **IV. TRANSCRIPTION** |  | **171** | **131-133** |
|  |  |  |  |  |
|  | ***rRNA transcription*** |  |  |  |
|  |  |  |  |  |
|  | ***rRNA synthesis*** |  | **4** | **4** |
| 22835, 24070 | RNA pol I, first largest sub (RPA1 SCHPO) | ECU04_1400 |  |  |
| 22527 | RNA pol I, second largest sub (RPA2 yeast) | ECU03_0440 |  |  |
| 26085, 27192 | RNA pol I, 40K sub (RPA5 HUMAN) | ECU01_0600 |  |  |
| 25540 | RNA pol I, 13.7K sub (RPA9 yeast | ECU08_1330 |  |  |
|  |  |  |  |  |
|  | ***rRNA processing*** |  | **20** | **17-18** |
| 25838 | ATP-dep RNA helicase (25S maturation), DEAD box fam (SPB4 yeast) | ECU10_0690 |  |  |
| 26693^1b^ | ATP-dep RNA helicase (P68-like), DEAD box fam (DBP2 yeast) | ECU08_1080 |  |  |
| 22204, 22357, 22982, 23171, 23284, 23411, 23785, 23888, 24119, 24159, 24899, 24924, 25286, 25295, 26319 | ATP-dep RNA helicase (25S maturation), put (YA47 SCHPO) | ECU06_1250 |  |  |
| 21820, 26163, 26693^1b^ | ATP-dep RNA helicase (18S maturation), put (YN21 CAEEL) | ECU10_0440 |  |  |
|  | belongs to the RNase PH (exosome) fam, put (YBX3 SCHPO) | ECU06_0390 |  |  |
|  | belongs to the RNase PH (exosome) fam, put (YG2N yeast) | ECU10_0270 |  |  |
| 24366 | component of the 3', 5' exoribonuclease for 3' end formation of 5.8S rRNA (DIS3 yeast) | ECU03_0700 |  |  |
| 22736 | component of the 3', 5' exoribonuclease for 3' processing of 5.8S rRNA (RNPH CAEEL) | ECU09_0830 |  |  |
| 26125 | fibrillarin (FBR2 yeast) | ECU10_0820 |  |  |
| 26118 | nucleolar prt (NOP2 yeast) | ECU07_0320 |  |  |
| 27494 | nucleolar prt, pre-18S splicing (NOP5 yeast) | ECU04_0820 |  |  |
| 25424 | nucleolar prt, pre-18S splicing (NOP5 yeast) | ECU09_1600 |  |  |
| 24867 | nucleolar prt, NOL1/NOP2 fam (p120 HUMAN) | ECU01_1080 |  |  |
| 26851 | rRNA adenine-N6,N6-dimethylTase (DIM1 yeast) | ECU04_0460 |  |  |
| 21790 | rRNA methylTase (SPB1 yeast) | ECU07_1340 |  |  |
| 27471 | small nucleolar prt (GAR1 yeast) | ECU01_1360 |  |  |
| 27169 | small nucleolar prt (GAR2 SCHPO) | ECU03_0730 |  |  |
| 23249 | U3 snoRNP, 18S production (IMP3 yeast) | ECU05_1320 |  |  |
| 24196 | U3 snoRNP, 18S production (SOF1 yeast) | ECU02_0240 |  |  |
| 27149 | U3 snoRNP, IMP4-like prt (YEE7 SCHPO) | ECU11_1910 |  |  |
|  |  |  |  |  |
|  | ***tRNA transcription*** |  |  |  |
|  |  |  |  |  |
|  | ***tRNA synthesis*** |  | **7** | **6** |
| 22530 | RNA pol III, 160K sub (RPC1 yeast) | ECU07_0090 |  |  |
| 26155 | RNA pol III, 130K sub (RPC2 SCHPO) | ECU11_0370 |  |  |
| 24426 | RNA pol III, 34K sub (RPC6 yeast) | ECU10_0210 |  |  |
|  | RNA pol III, 25K sub (RPCY yeast) | ECU08_0650 |  |  |
| 26246 | RNA pol III, 12.5K sub (RPCZ yeast) | ECU03_0490 |  |  |
| 27693 | RNA pol I and III, 14K sub (RPC9 HUMAN) | ECU11_0590 |  |  |
| 22687 | transcription init factor TFIIIB, 70K sub (TF3B yeast) | ECU05_0500 |  |  |
|  |  |  |  |  |
|  | ***tRNA processing*** |  | **2** | **2** |
| 24310 | ribonuclease P sub (RP29 HUMAN) | ECU07_1650 |  |  |
| 26266, 27199 | tRNA intron endonuclease (ENDA METTH | ECU03_0920 |  |  |
|  |  |  |  |  |
|  | ***tRNA modification*** |  | **7** | **7** |
| 26122 | N2,N2-dimethylguanosine-tRNA methylTase (TRM1 yeast) | ECU08_1450 |  |  |
| 23089, 25475, 25477, 25899, 26725, 26946, 27592, 27643 | tRNA pseudouridylate Sase 1(TRUA METTH) | ECU11_1610 |  |  |
| 21979 | tRNA pseudouridylate Sase 2 (PSU2 yeast) | ECU04_0890 |  |  |
| 26390 | tRNA pseudouridylate Sase 2 (TRUB METJA) | ECU05_1350 |  |  |
| 27076 | tRNA (Gm34, Gm37) 2'-O-methyltransferase (EC 2.1.1.34) | ECU09_0920 |  |  |
| 26373 | tRNA adenylylTrase (CCA1 yeast) | ECU03_1440 |  |  |
| 27162 | tRNA ribosylTase (TGT DROME) | ECU06_0310 |  |  |
|  |  |  |  |  |
|  | ***mRNA transcription*** |  |  |  |
|  |  |  |  |  |
|  | ***General activities*** |  |  |  |
|  |  |  |  |  |
|  | *DNA directed RNA polymerase II* |  | **12** | **10** |
| 22514 | RNA pol II, first sub (RPB1 SCHPO) | ECU03_0290 |  |  |
| 25876 | RNA pol II, second sub (RPB2 SCHPO) | ECU10_0250 |  |  |
| 27192 | RNA pol II, third sub (RPB3 SCHPO) | ECU06_0600 |  |  |
| 27702 | RNA pol II, 19K sub (RPB7 HUMAN) | ECU07_1750 |  |  |
|  | RNA pol II, 16K sub (RPB4 HUMAN) | ECU05_1060 |  |  |
| 25980 | RNA pol II, 16K sub (RPB4 HUMAN) | ECU08_0765 |  |  |
| 22564 | RNA pol II, 14.5K sub (RPB9 HUMAN) | ECU11_1400 |  |  |
| 25640, 26507, 26789 | RNA pol II, 14.1K sub (RPBY SCHPO) | ECU05_0275 |  |  |
| 24355 | RNA pol II, 8.2K sub (RPBX SCHPO) | ECU06_0320 |  |  |
| 25862 | RNA pol I, II and III, 27K sub (RPB5 yeast) | ECU07_0960 |  |  |
| 26001, 26036 | RNA pol I, II and III, 23K sub (RPB6 yeast) | ECU08_0320 |  |  |
|  | RNA pol I, II and III, 17K sub (RPB8 CAEEL) | ECU07_0840 |  |  |
|  |  |  |  |  |
|  | *Transcription initiation factors* |  | **18** | **17** |
| 23181, 23542, 24135, 24505, 24854, 25005, 25131, 25184, 26608, 27409, 27606 | TFIIA, γ sub (T2AG ARATH) | ECU02_0450 |  |  |
| 22131, 23009, 23379, 23415, 23910, 24020, 24951, 26262, 27617, | TFIIB (TF2B XENLA) | ECU06_1100 |  |  |
|  | TFIIB-related prt (SS72 yeast) | ECU06_0840 |  |  |
| 26482 | TFIID-1, TBP (TF2D ACACA) | ECU04_1440 |  |  |
| 24657 | TFIID, 150K sub (T2D2 DROME) | ECU09_0090 |  |  |
| 22821 | TFIID, 111K sub (T111 SCHPO) | ECU10_0760 |  |  |
| 24317, 26560 | TFIID, 72K sub (T2D4 SCHPO) | ECU11_1750 |  |  |
| 26119 | TFIID, 70K sub (T2D5 yeast) | ECU10_1120 |  |  |
| 22584 | TFIID, 30K sub (T2D8 HUMAN) | ECU11_0830 |  |  |
| 26414 | TFIID, 28K sub (T2D9 HUMAN) | ECU02_1250 |  |  |
| 26073 | TFIID, 18K sub (T2DB yeast) | ECU04_0950 |  |  |
| 23123 | TFIIE, α sub (T2EA HUMAN) | ECU07_0220 |  |  |
| 24137, 26981 | TFIIE, β sub (T2EB HUMAN) | ECU06_0350 |  |  |
| 26458 | TFIIF, β sub (T2FB HUMAN) | ECU02_0780 |  |  |
| 27448 | TFIIF, small sub, put (YD67 SCHPO) | ECU03_1600 |  |  |
| 27691 | TFIIH, Tfb sub (MAT1 HUMAN) | ECU11_0220 |  |  |
| 27427 | transcription init factor (SPT4 HUMAN) | ECU08_1660 |  |  |
| 27472 | transcription init factor, put (YAG5 SCHPO)-TAF9 | ECU01_1330 |  |  |
|  |  |  |  |  |
|  | *Transcription elongation factors* |  | **3** | **3** |
| 26369 | transcription elong factor SII (TFS2 SCHPO) | ECU04_0180 |  |  |
| 21994 | transcription elong factor, E2F/DP fam (E2F5 RAT) | ECU02_1350 |  |  |
| 26398 | transcription factor (dimerization partner), E2F/DP fam (TDP DROME) | ECU06_0260 |  |  |
|  |  |  |  |  |
|  | ***Transcriptional control*** |  | **44** | **34-36** |
| 23233, 26463 | antagonizes telomeric silencing (SIF2 yeast) | ECU01_0980 |  |  |
| 25550 | anti-silencing prt 1 (ASF1 yeast) | ECU07_0130 |  |  |
| 22918 | carbon catabolite repressor 4 (CCR4 yeast) | ECU11_0770 |  |  |
| 24429 | CCAAT-binding transcription factor, sub A (CBFA MAIZE) | ECU10_0260 |  |  |
| 22688 | CCAAT-binding factor sub (HAP5 yeast) | ECU05_0450 |  |  |
| 25272 | CCR4-associated transcription factor (CAF1 MOUSE) | ECU08_0850 |  |  |
| 26478 | GAL4 DNA-binding enhancer prt 2 (EGD2 yeast) | ECU02_1050 |  |  |
| 25391^1b^ | GATA-type transcription factor (GAF1 SCHPO) | ECU08_1400 |  |  |
| 25391^1b^ | GATA zinc-finger transcription factor 3 (GAT1 HUMAN) | ECU02_0260 |  |  |
| 22871 | general negative regulator of transcription, sub 1 (NOT1 yeast) | ECU11_1370 |  |  |
| 22333 | general negative regulator of transcription, sub 2 (NOT2 yeast) | ECU01_1040 |  |  |
| 22203, 22666, 23351, 23932, 24120, 24280, 24497, 24990, 26218 | general transcription factor (BDF1 yeast) | ECU08_1640 |  |  |
| 22811, 27356 | general transcriptional adaptator (ADA2 yeast) | ECU04_0230 |  |  |
| 23268 | global transcriptional activator, SNF2/RAD54 fam (SN21 HUMAN) | ECU09_1890 |  |  |
| 23973^1b^ | heat-shock transcription factor (HSF KLULA) | ECU04_0400 |  |  |
| 23973^1b^ | heat-shock transcription factor (HSF2 LYCPE) | ECU08_0970 |  |  |
| 23220 | histone deacetylase 1 (HDA1 CHICK) | ECU03_1370 |  |  |
| 24594 | histone deacetylase 3 (HDA3 CHICK) | ECU09_0670 |  |  |
| 27208 | histone transcription regulator (HIRL SCHPO) | ECU02_0710 |  |  |
| 24339 | removes TBP from the TATA box, SNF2/RAD54 fam (MOT1 yeast) | ECU03_1530 |  |  |
| 22128, 23195, 23302, 23706, 24303, 24587, 25594, 25648, 26580, 26668, 27138, 27576, 27633 | ring zinc finger transcriptional negative regulator (YAC4 SCHPO) | ECU11_1660 |  |  |
| 26470 | similarity to enhancer of zeste prt (EZ DROME) | ECU09_1750 |  |  |
| 25852 | similarity to suppressor of forked prt (SUF DROME) | ECU02_0930 |  |  |
|  | SIR2-like silencing prt (HST4 yeast) | ECU03_0460 |  |  |
| 25271 | TAU-like transcription factor (TFC4 yeast) | ECU08_0840 |  |  |
| 23074, 26279 | TBP-associated phosphoprotein (TBAP HUMAN) | ECU11_0730 |  |  |
| 24318 | transcription factor (STE2 CANAL) | ECU11_2030 |  |  |
| 27500^1b^ | transcription factor (FKH2 yeast) | ECU01_0470 |  |  |
| 22566, 27500^1b^ | transcription factor (FXJ1 HUMAN) | ECU11_1330 |  |  |
| 27143 | transcription factor, MYB-type (MYBH DICDI) | ECU06_1020 |  |  |
| 25858 | transcription factor, MYB-type (BAS1 yeast) | ECU05_0350 |  |  |
|  | transcription factor, put (SRF XENLA) | ECU07_1740 |  |  |
|  | transcription factor, TALE/PBX fam (HM20 CAEEL) | ECU10_1480 |  |  |
| 24936 | transcriptional activator (BTE1 HUMAN) | ECU05_0610 |  |  |
| 22717 | transcriptional activator (GCN5 yeast) | ECU10_1430 |  |  |
| 21706, 27499 | transcriptional activator (LMXA MESAU) | ECU03_1170 |  |  |
| 22311 | transcriptional regulation mediator (MED6 yeast) | ECU01_0500 |  |  |
|  | transcriptional regulator (BR14 HUMAN) | ECU09_1850 |  |  |
| 26147 | transcriptional regulator-like prt (SIN3 yeast) | ECU11_0190 |  |  |
|  | transcriptional repressor (TUP1 CANAL) | ECU02_0640 |  |  |
| 22682 | transcriptional repressor (NCB1 yeast) | ECU11_1470 |  |  |
| 22913^1b^, 26175^1b^ | zinc finger prt (GLO3 yeast) | ECU08_1690 |  |  |
| 22913^1b^, 26175^1b^ | zinc finger prt (GLO3 yeast) | ECU11_0550 |  |  |
| 27008 | zinc finger prt involved in transcriptional regulation (Z229 HUMAN) | ECU03_0790 |  |  |
|  |  |  |  |  |
|  | ***mRNA processing (splicing)*** |  |  |  |
|  |  |  |  |  |
|  | *Pre-mRNA splicing factors* |  | **9** | **4-6** |
|  | arginine/serine-rich pre-mRNA splicing factor (SFR4 HUMAN) | ECU05_1440 |  |  |
| 26693 | ATP-dep RNA helicase (DBP1 yeast) | ECU05_1270 |  |  |
| 24379^1b^ | ATP-dep RNA helicase, put (AN3 XENLA) | ECU02_0670 |  |  |
| 26402 | involved in pre-mRNA splicing (TR2H HUMAN) | ECU06_0230 |  |  |
| 22832^1b^, 23252^1b^ | pre-mRNA splicing factor (PR43 yeast) | ECU10_0680 |  |  |
| 22832^1b^ | pre-mRNA splicing factor (PRH1 SCHPO) | ECU04_0250 |  |  |
| 22832^1b^, 23252^1b^ | pre-mRNA splicing factor (PRH1 SCHPO) | ECU09_0150 |  |  |
| 22832^1b^, 23252^1b^ | pre-mRNA splicing factor (DDX8 SCHPO) | ECU11_0860 |  |  |
| 21911 | pre-mRNA splicing factor, put (BRR2 yeast) | ECU06_0920 |  |  |
|  |  |  |  |  |
|  | *U1 and U2 snRNPs* |  | **9** | **0** |
|  | splicing factor 3A-like prt sub (SP62 MOUSE) | ECU03_0480 |  |  |
|  | splicing protein for U2 snRNP binding to pre-mRNA (S145 HUMAN) | ECU11_1490 |  |  |
|  | U1 snRNP A (RU1A HUMAN) | ECU03_0750 |  |  |
|  | U1 snRNP C (RU1C MOUSE) | ECU10_0290 |  |  |
|  | U1/U2 snRNP (SMD1 CAEEL) | ECU01_0545 |  |  |
|  | U1/U2 snRNP (SMD2 yeast) | ECU10_1580 |  |  |
|  | U1/U2 snRNP G (RUXG SCHPO) | ECU05_0910 |  |  |
|  | U2 snRNP AF, 59K sub (U2AF MOUSE) | ECU05_0720 |  |  |
|  | U2 snRNP AF, 38K sub (U2AG DROME) | ECU09_1420 |  |  |
|  |  |  |  |  |
|  | *Other snRNPs* |  | **12** | **4** |
|  | snRNP D3 (SMD3 yeast) | ECU03_0840 |  |  |
|  | snRNP E, Sm fam (RUXE yeast) | ECU02_1020 |  |  |
|  | snRNP F, Sm fam (RUXF DROME) | ECU04_0790 |  |  |
|  | U5 snRNP-associated prt (MUQ1 yeast) | ECU11_0870 |  |  |
|  | U5 snRNP, PRP8 factor (PRO8 yeast) | ECU04_0760 |  |  |
| 26471 | U6 snRNP, Sm fam (SNP3 yeast) | ECU09_1805 |  |  |
|  | U6 snRNP, Sm fam, put (RUXX METTH) | ECU08_0135 |  |  |
|  | U6 snRNP, Sm fam, put (RUXX THEAS)^2^ | ECU07_1830 |  |  |
|  | U6 snRNP, Sm fam, put (RUXX THEAS)^2^ | ECU11_2070 |  |  |
| 23970 | U6 snRNP, Sm-like prt (LSM4 HUMAN) | ECU03_0360 |  |  |
| 25835 | U6 snRNP, Sm-like prt (LSM6 HUMAN) | ECU06_1140 |  |  |
| 26388 | U6 snRNP, Sm-like prt (LSM8 HUMAN) | ECU05_1310 |  |  |
|  |  |  |  |  |
|  | ***mRNA processing (5'-end, 3'-end processing)*** |  | **11** | **11** |
| 21793 | cleavage and polyadenylation specificity factor, 160K sub (CPSA HUMAN) | ECU11_0610 |  |  |
| 22846, 22910 | cleavage and polyadenylation specificity factor, 100K sub (CPSB BOVIN) | ECU05_0390 |  |  |
| 23159, 23211, 23431, 26030 | cleavage stimulation factor (CST2 HUMAN) | ECU08_0380 |  |  |
| 23224 | mRNA capping enzyme, sub alpha (MCE1 SCHPO) | ECU09_0400 |  |  |
| 27242 | mRNA capping enzyme, guanine N7 methylTase (ABD1 yeast) | ECU10_0380 |  |  |
| 25380 | mRNA decapping enzyme (DCP2 yeast) | ECU07_1630 |  |  |
| 22819 | polyadenylate-Bprt 1 (PABP SCHPO) | ECU07_0340 |  |  |
| 26027 | polyadenylate-Bprt 2 (PABP yeast) | ECU08_0520 |  |  |
| 22819 | polyadenylate-Bprt 2 (PABP SCHPO) | ECU10_1110 |  |  |
| 21701 | poly(A) polymerase (PAP yeast) | ECU02_0730 |  |  |
| 24593 | RNA trafficking prt (poly A degradation) (DHP1 SCHPO) | ECU09_0760 |  |  |
|  |  |  |  |  |
|  | ***Other*** |  | **5** | **3-4** |
| 21911 | RNA helicase (HFM1 yeast) | ECU07_0830 |  |  |
| 22788^1b^ | RNA helicase (SKI2W HUMAN) | ECU04_1290 |  |  |
| 24379^1b^ | RNA helicase, put (YA88 SCHPO) | ECU07_1060 |  |  |
| 22788^1b^ | RNA helicase, put (YDGV SCHPO) | ECU04_0910 |  |  |
| 24359 | RNA helicase, put (YP93 CAEEL) | ECU05_0940 |  |  |
|  |  |  |  |  |
|  | ***RNA transport*** |  | **5** | **4** |
| 25449 | mRNA-associated prt (RAE1 SCHPO) | ECU06_1550 |  |  |
| 24367 | nuclear cap-Bprt (CB20 HUMAN) | ECU03_0760 |  |  |
| 24596 | RNA-binding LA protein (LAH1 SCHPO) | ECU09_0680 |  |  |
|  | RNA-Bprt (YIS1 yeast) | ECU01_0540 |  |  |
| 23432, 23525, 24304, 25012, 25154, 25960, 26821, 27625 | RNA-Bprt, pumilio family (YDHA SCHPO) | ECU11_1730 |  |  |
|  |  |  |  |  |
|  | ***Other transcription activities*** |  | **3** | **2** |
|  | RNA helicase, DEAD box fam (HAS1 yeast) | ECU10_1680 |  |  |
| 21820, 25868, 26163, 26693 | RNA helicase, put (ST13 SCHPO) | ECU09_1640 |  |  |
| 23413, 25802, 26009, 26509, 26652, 26732, 26749, 26873, 26977 | RNA 3' terminal P cyclase (RTC1 SCHPO) | ECU06_1030 |  |  |
|  |  |  |  |  |
|  | **V. PROTEIN SYNTHESIS** |  | **123** | **112** |
|  |  |  |  |  |
|  | ***Ribosomal proteins*** |  |  |  |
|  |  |  |  |  |
|  | ***60S ribosomal proteins*** |  | **46** | **43-44** |
| 24198 | acidic ribosomal prt P0 (A0) (RLA0 yeast) | ECU07_0820 |  |  |
| 22684 | acidic ribosomal prt P0, w.sim (RLA0 ARCFU) | ECU05_0470 |  |  |
| 25553 | acidic ribosomal prt P2 (A4) (RLA4 SCHPO) | ECU07_0110 |  |  |
| 25999 | ribosomal prt L44 (RL44 ARATH) | ECU10_1300 |  |  |
|  | ribosomal prt L39 (RL39 DROME) | ECU09_0395 |  |  |
| 27696 | ribosomal prt L37 (RL37 SCHMA) | ECU07_1460 |  |  |
| 27681 | ribosomal prt L37A (L43) (R37A PLAFA) | ECU07_1005 |  |  |
| 26324, 27269, 27273, 27512 | ribosomal prt L36 (R36A yeast) | ECU06_1120 |  |  |
| 27698^1a^ | ribosomal prt L35 (RL35 SCHPO)^2^ | ECU07_1820 |  |  |
| 27698^1a^ | ribosomal prt L35 (RL35 SCHPO)^2^ | ECU11_2060 |  |  |
| 27698^1a^ | ribosomal prt L35, w.sim (RL35 SCHPO)^2^ | ECU10_0070 |  |  |
| 27683 | ribosomal prt L35A (L33) (R35A CAEEL) | ECU02_0900 |  |  |
| 27705 | ribosomal prt L34 (RL34 AEDAL) | ECU03_0710 |  |  |
| 27689 | ribosomal prt L32 (RL32 HUMAN) | ECU04_1310 |  |  |
| 26048, 27347 | ribosomal prt L31 (RL31 yeast) | ECU03_0230 |  |  |
| 27154 | ribosomal prt L30 (RL30 MAIZE) | ECU05_1490 |  |  |
| 23218 | ribosomal prt L27 (RL27 SOLTU) | ECU04_0330 |  |  |
| 26136 | ribosomal prt L27A (L28) (R27A ENCCU) | ECU10_0990 |  |  |
| 26031, 26640 | ribosomal prt L26 (RL26 CHICK) | ECU08_0370 |  |  |
| 24877 | ribosomal prt L24 (RL24 CAEEL) | ECU02_0810 |  |  |
| 24203 | ribosomal prt L24 (RL24 KLULA) | ECU07_0930 |  |  |
| 26460 | ribosomal prt L23 (RL23 HUMAN) | ECU08_1160 |  |  |
| 24442^1b^ | ribosomal prt L23A (L25) (RL2B FRIAG) | ECU10_0160 |  |  |
| 24442^1b^ | ribosomal prt L23A (L25) (RL23 PYRHO) | ECU08_1910 |  |  |
| 25842 | ribosomal prt L22 (RL22 TRIGR) | ECU04_0740 |  |  |
| 24363 | ribosomal prt L21 (RL21 CAEEL) | ECU05_0900 |  |  |
| 23416, 23641, 23911, 24363, 26005, 26229, 26934 | ribosomal prt L19 (RL19 MOUSE) | ECU06_1080 |  |  |
| 24312 | ribosomal prt L18 (RL18 XENLA) | ECU03_1490 |  |  |
| 25983 | ribosomal prt L18A (L20) (RL20 yeast) | ECU08_0600 |  |  |
| 25436, 25631, 26426, 26631, 26833, 27226, 27506, 27588 | ribosomal prt L17 (RL17 RAT) | ECU07_1410 |  |  |
| 25874 | ribosomal prt L15 (RL15 CHITE) | ECU11_1380 |  |  |
| 25837 | ribosomal prt L13 (RL13 CANAL) | ECU03_0320 |  |  |
| 26113 | ribosomal prt L13A (L16) (R16C SCHPO) | ECU04_1380 |  |  |
| 26143, 27289 | ribosomal prt L12 (RL12 SCHPO) | ECU08_2010 |  |  |
| 26857 | ribosomal prt L11 (RL11 DROME) | ECU02_0610 |  |  |
| 25406 | ribosomal prt L10 (RL10 HUMAN) | ECU08_1570 |  |  |
| 24935 | ribosomal prt L10A (L1) (R10A RAT) | ECU05_0600 |  |  |
| 27460 | ribosomal prt L9 (RL9 RAT) | ECU02_0800 |  |  |
| 22342, 24846 | ribosomal prt L8 (L2) (RL2 yeast) | ECU01_0310 |  |  |
| 21711 | ribosomal prt L7 (RL7C SCHPO) | ECU03_0950 |  |  |
| 27428 | ribosomal prt L7A (L8) (RL8B yeast) | ECU02_0750 |  |  |
| 22343 | ribosomal prt L6 (RL6A yeast) | ECU08_1780 |  |  |
| 22761 | ribosomal prt L5 (RL5 CAEEL) | ECU06_0900 |  |  |
| 22586, 22627, 22880, 23599, 24136, 25113, 25753, 25781, 25910, 25973, 27371 | ribosomal prt L4 (RL4 TRYBB) | ECU08_0830 |  |  |
| 21726, 24032^1b^ | ribosomal prt L3 (RL3 RAT) | ECU03_1220 |  |  |
| 21726, 24032^1b^ | ribosomal prt L3 (RL3 yeast) | ECU09_1000 |  |  |
|  |  |  |  |  |
|  | ***40S ribosomal proteins*** |  | **31** | **26** |
| 25663 | ribosomal prt S30 (RS30 yeast) | ECU10_1575 |  |  |
| 27707 | ribosomal prt S29 (RS29 HUMAN) | ECU04_0125 |  |  |
| 27706 | ribosomal prt S28 (RS28 ARATH) | ECU09_1275 |  |  |
| 27687 | ribosomal prt S27 (RS27 XENLA) | ECU04_1015 |  |  |
|  | ribosomal prt S26 (RS26 NEUCR) | ECU06_1445 |  |  |
|  | ribosomal prt S25 (RS25 yeast)^2^ | ECU08_1040 |  |  |
|  | ribosomal prt S25 (RS25 yeast)^2^ | ECU08_1070 |  |  |
| 25693, 26190, 27296, 27516 | ribosomal prt S24 (RS24 FUGRU) | ECU10_1570 |  |  |
| 27241 | ribosomal prt S23 (RS23 BRUMA) | ECU10_0400 |  |  |
| 26287, 26296 | ribosomal prt S20 (RS20 ORYSA) | ECU11_0720 |  |  |
| 25476, 25771, 25898, 26760 | ribosomal prt S19 (R19E METJA) | ECU11_1620 |  |  |
| 22181, 23784, 25952, 27616 | ribosomal prt S18 (RS18 HUMAN) | ECU06_1110 |  |  |
| 27425 | ribosomal prt S17 (RS17 HUMAN) | ECU02_0770 |  |  |
| 25836 | ribosomal prt S16 (RS16 LUPPO) | ECU03_0310 |  |  |
| 23929, 24150, 26034 | ribosomal prt S15 (RS15 ARATH) | ECU08_0350 |  |  |
| 24033, 23752, 24726, 25368, 25626, 25738, 25758, 26199, 26212, 26898, 27300, 27342 | ribosomal prt S15A (S22) (RS22 yeast) | ECU09_1350 |  |  |
| 27704 | ribosomal prt S14 (RS14 SCHPO) | ECU03_0650 |  |  |
|  | ribosomal prt S13 (RS13 BRUPA) | ECU08_1060 |  |  |
|  | ribosomal prt S12 (RS12 XENLA) | ECU01_0920 |  |  |
| 26379 | ribosomal prt S11 (RS11 yeast) | ECU04-0640 |  |  |
| 27690 | ribosomal prt S10 (RS10 SCHPO) | ECU04_1355 |  |  |
| 27165 | ribosomal prt S9 (RS9 CAEEL) | ECU05_0920 |  |  |
| 25867 | ribosomal prt S8 (RS8 SCHPO) | ECU02_0880 |  |  |
| 22919 | ribosomal prt S7 (RS7 SCHPO) | ECU11_0780 |  |  |
| 27434 | ribosomal prt S6 (RS6 KLULA) | ECU05_0670 |  |  |
| 27464 | ribosomal prt S5 (RS5 DROME) | ECU04_0140 |  |  |
| 25278 | ribosomal prt S4 (RS4 GOSHI) | ECU08_0870 |  |  |
| 21810 | ribosomal prt S3 (RS3 yeast) | ECU09_1250 |  |  |
| 21835, 21946, 22052, 24415, 24607, 25532, 25605, 26294, 26529, 26536, 26681, 26910, 26970, 26994, 27299, 27366, | ribosomal prt S3A (S1) (RS3A ORYZA) | ECU05_0250 |  |  |
| 26061 | ribosomal prt S2 (RS2 MOUSE) | ECU07_1700 |  |  |
| 22833 | ribosomal prt SA or P40 (S0) (RSP4 MOUSE) | ECU04_0450 |  |  |
|  |  |  |  |  |
|  | ***Translation*** |  |  |  |
|  |  |  |  |  |
|  | ***Initiation*** |  | **12** | **11** |
| 24658 | translation init factor eIF2, α sub (IF2A METJA) | ECU11_0930 |  |  |
| 24672 | translation init factor eIF2, β sub (IF2B yeast) | ECU06_0210 |  |  |
| 22791 | translation init factor eIF2, γ sub (IF2G ENCCU) | ECU01_0700 |  |  |
|  | translation init factor eIF2B, γ sub (E2BG CAEEL) | ECU05_1360 |  |  |
| 24659 | translation init factor eIF2B, ε sub (E2B2 yeast) | ECU10_0700 |  |  |
| 27253 | translation init factor eIF3, β sub (IF32 HUMAN) | ECU10_0220 |  |  |
| 21820 | translation init factor eIF4A (IF4A yeast) | ECU09_1200 |  |  |
| 25445 | translation init factor eIF4E (IF4E XENLA) | ECU08_0900 |  |  |
| 22569 | translation init factor eIF4E (IF4E APLCA) | ECU11_1350 |  |  |
| 21780 | translation init factor eIF5, EIF2B/EIF5 fam, (IF5 SCHPO) | ECU07_133 |  |  |
| 26411 | translation init factor eIF6 (IF6 HUMAN) | ECU04_0780 |  |  |
| 27095 | eIF-1A | ECU04_1170 |  |  |
|  |  |  |  |  |
|  | ***Elongation*** |  | **5** | **4** |
| 22121, 23902, 23947, 24297, 24994, 25238, 26432, 26630, 26755, 26780, 27001, 27006, 27228, 27507, 27584 | translation elong factor eEF1α (EF1A ENTHI) | ECU06_1440 |  |  |
| 24216 | translation elong factor eEF1α (EF1A MAIZE) | ECU04_1100 |  |  |
| 26306 | translation elong factor eEF2 (EF2 METTH) | ECU11_1120 |  |  |
| 22563, 22966 | translation elong factor eEF2 (EF2 SCHPO) | ECU11_1460 |  |  |
|  | translation elong factor EF-G, bacterial type (EFG BACSU) | ECU09_0810 |  |  |
|  |  |  |  |  |
|  | ***Termination*** |  | **2** | **2** |
| 21803 | peptide chain release factor (ERF1 XENLA) | ECU05_0360 |  |  |
| 26418 | ribosome recycling factor (RRF THEMA) | ECU01_0960 |  |  |
|  |  |  |  |  |
|  | ***Other*** |  | **1** | **1** |
| 23859, 23869 | translation init factor IF2P, bacterial type (IF2P yeast) | ECU09_0070 |  |  |
|  |  |  |  |  |
|  | ***Translational control*** |  | **5** | **3** |
|  | involved in mRNA decay control (NAM7 yeast) | ECU10_1640 |  |  |
| 24333 | nonsense-mediated mRNA decay prt (NMD3 yeast) | ECU09_1380 |  |  |
| 22906 | nonsense-mediated mRNA decay prt (NMD5 yeast) | ECU10_0620 |  |  |
| 22830, 26095, 26368 | suppressor of stem-loop (SSL1 yeast) | ECU04_0280 |  |  |
|  | translational activator, put (YAQ5 SCHPO) | ECU07_0140 |  |  |
|  |  |  |  |  |
|  | ***tRNA synthetases*** |  | **21** | **21** |
| 22575 | alanyl-tRNA Sase (SYAC yeast) | ECU02_1490 |  |  |
| 22720 | arginyl-tRNA Sase (SYRC yeast) | ECU08_0550 |  |  |
| 22533 | asparaginyl-tRNA Sase (SYNC yeast) | ECU09_1680 |  |  |
| 21705 | aspartyl-tRNA Sase (SYD RAT) | ECU06_0790 |  |  |
| 21788 | cysteinyl-tRNA Sase (SYC PYRHO) | ECU08_0490 |  |  |
| 22570 | glutaminyl-tRNA Sase (SYQ LUPLU) | ECU10_1460 |  |  |
| 22577 | glutamyl-tRNA Sase (SYEC yeast) | ECU02_1210 |  |  |
| 25557 | glycyl-tRNA Sase (SYG HUMAN) | ECU10_1790 |  |  |
| 23237 | histidyl-tRNA Sase (SYH BORBU) | ECU06_0620 |  |  |
| 22873 | isoleucyl-tRNA Sase (SYI HUMAN) | ECU11_1100 |  |  |
| 21702, 24031 | leucyl-tRNA Sase (SYLC SCHPO) | ECU06_0280 |  |  |
| 25548 | lysyl-tRNA Sase (SYK CRILO) | ECU04_0580 |  |  |
| 21922, 22150, 22625, 22784, 23412, 24001, 24314, 24510, 24585, 24634, 25120, 25262, 25303, 25471, 25635, 25751, 26739, 26903, 27118, 27261, 27303, 27340 | methionyl-tRNA Sase (SYM CAEEL) | ECU11_0890 |  |  |
| 22775 | phenylalanyl-tRNA Sase, α sub (SYFA HUMAN) | ECU07_1660 |  |  |
| 21956 | phenylalanyl-tRNA Sase, β sub (SYFB MOUSE) | ECU04_0900 |  |  |
| 21971 | prolyl-tRNA Sase (SYEP DROME) | ECU02_1360 |  |  |
| 22713 | seryl-tRNA Sase (SYS HELAN) | ECU04_0750 |  |  |
| 22768 | threonyl- tRNA Sase (SYTC yeast) | ECU07_1570 |  |  |
| 22423, 22597, 23527, 23817, 25071, 26333, 27396 | tryptophanyl-tRNA Sase (SYW SCHPO) | ECU11_0530 |  |  |
| 24879 | tyrosyl-tRNA Sase (SYY HUMAN) | ECU05_1120 |  |  |
| 24206 | valyl-tRNA Sase (SYV yeast) | ECU04_1140 |  |  |
|  |  |  |  |  |
|  | **VI. PROTEIN DESTINATION** |  | **123** | **101-105** |
|  |  |  |  |  |
|  | ***protein folding and stabilization*** |  |  |  |
|  |  |  |  |  |
|  | ***T-complex protein 1 (TCP1)*** |  | **8** | **8** |
| 23981 | TCP1, alpha sub (TCPA yeast) | ECU03_0220 |  |  |
| 23226 | TCP1, beta sub (TCPB HUMAN) | ECU09_0480 |  |  |
| 24428 | TCP1, gamma sub (TCPG yeast) | ECU10_0240 |  |  |
| 24220 | TCP1, delta sub (TCPD HUMAN) | ECU02_0520 |  |  |
| 22350, 22746, 22757, 23616, 23686, 24093, 24383 | TCP1, epsilon sub (TCPE HUMAN) | ECU06_0990 |  |  |
| 22904 | TCP1, eta sub (TCPH SCHPO) | ECU10_0630 |  |  |
| 26072 | TCP1, theta sub (TCPQ yeast) | ECU04_1020 |  |  |
| 27459 | TCP1, zeta sub (TCPZ HUMAN) | ECU06_0510 |  |  |
|  |  |  |  |  |
|  | ***Other chaperones*** |  | **8** | **6** |
| 27439 | chaperone HSB (HSC20) (HSBC HAEIN) | ECU02_0690 |  |  |
|  | DNAJ-like prt (DNAJ THEMA) | ECU01_0570 |  |  |
|  | DNAJ-like prt (DNAJ CHLTR) | ECU10_1100 |  |  |
| 24309 | heat-shock prt, HSP70 fam (HS70 PLAFA) | ECU11_1830 |  |  |
| 22534 | heat-shock prt, HSP70 fam (HS7C MOUSE) | ECU03_0520 |  |  |
| 22915 | heat-shock prt, HSP70 fam, mito (GR75 HUMAN) | ECU11_0540 |  |  |
| 21949 | heat-shock prt HSP90 (HS9 BRARE) | ECU02_1100 |  |  |
| 21720 | HS70 prt cognate 4 (HSC70) (HS72 yeast) | ECU02_0100 |  |  |
|  |  |  |  |  |
|  | ***Isomerases*** |  | **5** | **4** |
|  | peptidyl prolyl cis-trans isomerase (PIN1 HUMAN) | ECU06_0330 |  |  |
| 21707 | peptidyl prolyl cis-trans isomerase (cyclophilin) (CYPH DROME) | ECU08_0470 |  |  |
| 22329 | peptidyl prolyl cis-trans isomerase (cyclophilin) (CYPH CATRO) | ECU10_1760 |  |  |
| 26685 | protein disulfide isomerase (PDI MEDSA) | ECU09_1560 |  |  |
| 25871 | protein disulfide isomerase (PDI1 SCHPO) | ECU02_0850 |  |  |
|  |  |  |  |  |
|  | ***Protein targeting, sorting and translocation*** |  |  |  |
|  |  |  |  |  |
|  | ***ER protein-translocation complex*** |  | **5** | **5** |
| 24666 | SEC61 homolog, α sub (S61A yeast) | ECU09_0130 |  |  |
| 27703 | SEC61 homolog, γ sub (S61G yeast) | ECU05_0885 |  |  |
| 27086 | SEC62 homolog (SC62 yeast) | ECU07_0700 |  |  |
| 26016 | SEC63 homolog (NPL1 yeast) | ECU06_0870 |  |  |
| 22795 | SEC66 homolog (SC66 yeast) | ECU04_0940 |  |  |
|  |  |  |  |  |
|  | ***Other*** |  | **6** | **6** |
| 24215 | involved in ER translocation (NPL4 yeast) | ECU07_0770 |  |  |
| 21804, 25452, 25453 | KDEL receptor (ERD2 DROME) | ECU06_1570 |  |  |
| 22787 | signal recognition particle (SRP) sub (SR54 yeast) | ECU04_0980 |  |  |
| 27684 | signal recognition particle (SRP) sub (SC65 KLULA) | ECU08_0800 |  |  |
| 27438 | signal sequence receptor, α sub (SSRP TREPA) | ECU04_1230 |  |  |
| 24431 | SRP receptor, α sub (SRPR HUMAN) | ECU10_0170 |  |  |
|  |  |  |  |  |
|  | ***Protein modification*** |  |  |  |
|  |  |  |  |  |
|  | ***Acetylation*** |  | **4** | **3** |
| 24200 | histone acetylTase type B, sub (RB46 HUMAN) | ECU07_0750 |  |  |
| 24599 | histone acetylTase (ESA1 yeast) | ECU10_0660 |  |  |
|  | N-terminal acylTase complex, ARD sub (ARDH HUMAN) | ECU06_0460 |  |  |
| 22774 | N-terminal acylTase complex, ARD sub (ARDH LEIDO) | ECU07_1680 |  |  |
|  |  |  |  |  |
|  | ***Glycosylation*** |  | **7** | **5** |
| 26419 | dolichyl-P-β-D-mannosylTase (DPM1 yeast) | ECU04_1060 |  |  |
| 27686 | dolichyl-P-mannose-protein O-mannosylTase (PMT1 CANAL) | ECU06_0950 |  |  |
| 24422 | dolichyl-P-mannose-protein O-mannosylTase (PMT3 yeast) | ECU02_1300 |  |  |
| 24671 | mannose-1-P guanylylTase (MPG1 yeast) | ECU11_0690 |  |  |
| 22536 | phosphomannomutase (PMM CANAL) | ECU05_0260 |  |  |
|  | phosphomanno(gluco)mutase (YML8 yeast) | ECU03_0340 |  |  |
|  | UDP N-acetylglucosamine peptide N-acetyl-glucosaminylTase, 100K sub (OGT1 RAT) | ECU08_1340 |  |  |
|  |  |  |  |  |
|  | ***Myristylation*** |  | **1** | **1** |
| 22377 | peptide N-myristoylTase (NMT1 MOUSE) | ECU03_1350 |  |  |
|  |  |  |  |  |
|  | ***Prenylation*** |  | **3** | **3** |
| 23267 | dehydrodolichyl diP Sase (cis-prenylTase) (RER2 yeast) | ECU09_1870 |  |  |
| 26479 | protein-farnesylTase (CAAX farnesylTase) α sub (RAM2 YEAST) | ECU02_1060 |  |  |
| 25547 | protein-geranylgeranylTase type II, β sub (BET2 yeast) | ECU08_1290 |  |  |
|  |  |  |  |  |
|  | ***Processing*** |  | **10** | **6-7** |
| 26392 | CAAX prenyl protease (ST24 yeast) | ECU05_1370 |  |  |
| 25397, 25575 | CAAX prenyl protease (ST24 yeast) | ECU02_1380 |  |  |
| 21985^1a^ | glutamyl-aminopeptidase (AMPE HUMAN)^2^ | ECU01_0140 |  |  |
| 21985^1a^ | glutamyl-aminopeptidase (AMPE HUMAN)^2^ | ECU01_1470 |  |  |
| 21985^1b^ | glutamyl-aminopeptidase (AMPE MOUSE) | ECU08_0070 |  |  |
| 27249 | methionine aminopeptidase 2 (MAP2 yeast) | ECU10_0750 |  |  |
| 25516 | signal peptidase, 21K sub (SPC3 CANFA) | ECU02_1140 |  |  |
| 27426 | signal peptidase, 18K sub (SPC4 CANFA) | ECU02_0760 |  |  |
|  | signal peptidase-like prt, bacterial type (TPL TREPA) | ECU06_1580 |  |  |
|  | similarity to mammalian microsomal signal peptidase sub (YJB2 yeast) | ECU06_1420 |  |  |
|  |  |  |  |  |
|  | ***Other*** |  | **3** | **3** |
| 23229 | gamma-glutamyl transpeptidase (GGT PIG) | ECU05_1240 |  |  |
| 25443 | guanosine diphosphatase (GDA1 yeast) | ECU07_1260 |  |  |
| 21713, 22789, 24213, 24928, 25561, 27474 | ribosomal protein S6 kinase (KS6 HUMAN) | ECU10_0570 |  |  |
|  |  |  |  |  |
|  | ***Proteolysis*** |  |  |  |
|  |  |  |  |  |
|  | ***Cytoplasmic degradation*** |  |  |  |
|  |  |  |  |  |
|  | *26S proteasome* |  | **31** | **30** |
| 22485, 23196, 25845, 26581, 26682, 27137, 27139 | proteasome alpha-type sub (PRC2 MOUSE) | ECU11_1670 |  |  |
| 21725 | proteasome alpha-type sub (PRC3 HUMAN) | ECU08_1580 |  |  |
|  | proteasome alpha-type sub, w.sim (PRC3 HUMAN) | ECU11_1840 |  |  |
| 23238 | proteasome alpha-type sub (PRC5 RAT) | ECU07_1420 |  |  |
| 21870, 23569, 24116, 25971, 26542, 27361, 27544 | proteasome alpha-type sub (PRC6 LYCES) | ECU07_1040 |  |  |
| 24421 | proteasome alpha-type sub (PRC6 HUMAN) | ECU10_0550 |  |  |
| 22849 | proteasome alpha-type sub (PRC8 yeast) | ECU09_0330 |  |  |
| 26389 | proteasome alpha-type sub (PRC9 yeast) | ECU05_1340 |  |  |
| 26393 | proteasome alpha-type sub (PRCZ DROME) | ECU05_1400 |  |  |
| 22127, 25637, 26322, 26828, 26950 | proteasome beta-type sub (PRCB MOUSE) | ECU05_0290 |  |  |
| 22716 | proteasome beta-type sub (PRCD yeast) | ECU10_1450 |  |  |
| 22334 | proteasome beta-type sub (PRCE yeast) | ECU08_1870 |  |  |
| 24595 | proteasome beta-type sub (PRCF yeast) | ECU09_0720 |  |  |
| 25860 | proteasome beta-type sub (PRCG RAT) | ECU08_0280 |  |  |
| 21688 | proteasome beta-type sub (PRCT yeast) | ECU02_0340 |  |  |
| 22363, 22469, 22509, 22595, 22624, 23408, 23577, 23793, 23883, 24155, 24182, 24626, 24741, 24771, 24985, 25057, 25478, 25674, 25708, 25767, 25775, 25790, 25918, 26316, 26615, 26703, 26922, 26948, 27007, 27408, 27547, 27609, 27640 | proteasome regul sub 2 (HRD2 yeast) | ECU02_0480 |  |  |
| 21814 | proteasome regul sub 3 (PSD3 HUMAN) | ECU05_1540 |  |  |
| 23243 | proteasome regul sub 4 (MTS4 SCHPO) | ECU04_0310 |  |  |
| 22766 | proteasome regul sub 4 (PRS4 CAEEL) | ECU07_1640 |  |  |
| 27239 | proteasome regul sub 5A (PSD4 ARATH) | ECU10_0410 |  |  |
| 24443^1b^ | proteasome regul sub 6 (PRS6 RAT) | ECU08_1970 |  |  |
| 24443^1b^ | proteasome regul sub 6 (PRS6 RAT) | ECU10_0130 |  |  |
| 22718 | proteasome regul sub 6A (TAT-binding prt 1) (PRSA ORYZA) | ECU10_1420 |  |  |
| 23971 | proteasome regul sub 7 (PRS7 HUMAN) | ECU03_1330 |  |  |
| 23277 | proteasome regul sub 8 (PRS8 SCHPO) | ECU09_1840 |  |  |
| 21983 | proteasome regul sub 10 (PRSX SPETR) | ECU07_0190 |  |  |
| 22916 | proteasome regul sub 11 (PAD1 SCHPO) | ECU11_0570 |  |  |
| 23967 | proteasome regul sub 12 (PRS12 ARATH) | ECU04_0420 |  |  |
| 22585, 22798, 22799, 25889, 25890, 26042, 26257 | proteasome regul sub YTA6, AAA fam of ATPases (TBP6 yeast) | ECU11_0840 |  |  |
| 22558 | proteasome regul sub YTA6, AAA fam of ATPases (TBP6 yeast) | ECU11_1030 |  |  |
| 24863 | protein of the CDC48/PAS1/SEC28 fam of ATPases (AAA) (CC48 ARATH) | ECU01_1230 |  |  |
|  |  |  |  |  |
|  | *Ubiquitin* |  | **3** | **2** |
| 21975 | monoubiquitin/carboxy-extension protein fusion (UBI1 CEP) | ECU02_1080 |  |  |
| 26439, 27429 | ubiquitin (UBIQ STRPV) | ECU02_0740 |  |  |
|  | ubiquitin-like prt (SMT3 SCHPO) | ECU07_0660 |  |  |
|  |  |  |  |  |
|  | *Ubiquitin carboxy-terminal hydrolase* |  | **4** | **4** |
| 26012 | ubiquitin C-terminal hydrolase (UBPB HUMAN) | ECU06_0910 |  |  |
| 25851 | ubiquitin C-terminal hydrolase (UBPB yeast) | ECU03_0580 |  |  |
| 25563, 26074 | ubiquitin C-terminal hydrolase (UBPC HUMAN) | ECU07_0410 |  |  |
| 24365 | ubiquitin C-terminal hydrolase (UBPE yeast) | ECU03_0660 |  |  |
|  |  |  |  |  |
|  | *Ubiquitin-activating enzyme E1* |  | **2** | **1** |
|  | similarity to C-terminal of ubiquitin-activating enzyme E1 (YA7C SCHPO) | ECU02_1340 |  |  |
| 21708, 23142 | ubiquitin-activating enzyme E1 (UBA1 yeast) | ECU03_0930 |  |  |
|  |  |  |  |  |
|  | *Ubiquitin-conjugating enzyme E2* |  | **8** | **5-6** |
| 27493^1b^, 26416^1b^ | ubiquitin-conjugating E2 sub (UBC1 HUMAN) | ECU01_0940 |  |  |
| 27145^1b^ | ubiquitin-conjugating E2 sub (UBCB yeast) | ECU10_1540 |  |  |
| 27493^1b^ | ubiquitin-conjugating E2 sub (UBC2 yeast) | ECU10_1310 |  |  |
| 27145^1b^ | ubiquitin-conjugating E2 sub (UBC5 yeast) | ECU11_1990 |  |  |
| 22843^1b^ | ubiquitin-conjugating E2 sub (UBC6 HUMAN) | ECU10_0940 |  |  |
| 25961 | ubiquitin-conjugating E2 sub (UBC7 ARATH) | ECU01_1010 |  |  |
| 26378 | ubiquitin-conjugating E2 sub (UBC7 CAEEL) | ECU04_0630 |  |  |
| 22843^1b^, 26416^1b^ | ubiquitin-conjugating E2 sub (UBC8 yeast) | ECU08_0860 |  |  |
|  |  |  |  |  |
|  | *Ubiquitin protein ligase E3* |  | **2** | **1-2** |
| 23965^1b^, 25782 | ubiquitin protein ligase E3A (UE3A HUMAN) | ECU04_0490 |  |  |
| 23965^1b^ | ubiquitin protein ligase E3, RSP5-type (RSP5 yeast) | ECU10_1380 |  |  |
|  |  |  |  |  |
|  | *Other* |  | **4** | **3** |
| 26116 | ubiquitin fusion and degradation prt 1 (UFD1 yeast) | ECU10_1180 |  |  |
|  | ubiquitin fusion and degradation prt 2 (UFD2 yeast) | ECU04_1210 |  |  |
| 26469 | WD-repeat protein involved in ubiquitin-dep proteolysis (DOA1 yeast) | ECU09_1740 |  |  |
| 25439 | Ubi/L40 fusion protein | ECU07_1380 |  |  |
|  |  |  |  |  |
|  | **Other** |  | **9** | **6** |
| 24193 | cytosol aminopeptidase (X-Leu / X-Pro), put (YA55 SCHPO) | ECU10_1770 |  |  |
|  | heat-shock protein H101, CLPB fam of ATP dep proteases (CLPB HAEIN) | ECU11_1420 |  |  |
| 22797 | O-sialoglycoprotein endopeptidase, put (YK18 yeast) | ECU09_0140 |  |  |
|  | peptidase, put (YJ96 CAEEL) | ECU09_1070 |  |  |
| 24883 | subtilisin-like serine protease (PEPC ASPNG) | ECU01_1130 |  |  |
|  | subtilisin-like serine protease (endopeptidase K) (PRTK TRIAL) | ECU03_1180 |  |  |
| 21957 | X-Pro dipeptidase (PEPQ LACHE) | ECU04_0600 |  |  |
| 27098 | zinc metallopeptidase (YE94 SCHPO) | ECU06_0380 |  |  |
| 21700 | zinc protease, insulinase fam (IDE DROME) | ECU06_0750 |  |  |
|  |  |  |  |  |
|  | **VII. TRANSPORT FACILITATION** |  | **40** | **19-23** |
|  |  |  |  |  |
|  | ***Ion transporters*** |  | **6** | **4** |
| 21689 | cation-transporting ATPase (ATC6 yeast) | ECU02_0330 |  |  |
| 26064, 26069, 26421 | cation-transporting ATPase (ATC9 yeast) | ECU07_1500 |  |  |
| 24564, 25769, 25924, 26065, 26224, 26715, 26752, 27282 | inorganic phosphate transp (PH88 yeast) | ECU11_1640 |  |  |
| 26451 | Na^+^/H^+^ antiporter (NHA1 yeast) | ECU04_1470 |  |  |
|  | Na^+^-transporting V-type ATPase, proteolipid component (K subunit) (NTPK NTHR) | ECU03_1120 |  |  |
|  | zinc transp (endosomes) (ZNT4 HUMAN) | ECU11_1510 |  |  |
|  |  |  |  |  |
|  | ***Sugar transporters*** |  | **3** | **2** |
| 24636^1b^ | glucose transp, type 1 (GTR1 PIG) | ECU07_1100 |  |  |
| 24636^1b^ | glucose transp, type 3 (GTR3 CANFA) | ECU04_0210 |  |  |
| 24311, 22442, 26550, 26877, 26902 | sugar permease (MALA BACST) | ECU11_1870 |  |  |
|  |  |  |  |  |
|  | ***Amino-acid and oligopeptide transporters*** |  | **7** | **1-2** |
| 26295^1b^ | amino-acid transp, put (YEH4 yeast) | ECU11_1740 |  |  |
| 21813^1b^ | amino-acid transp, put (YEU9 yeast) | ECU04_1190 |  |  |
| 21813^1b^, 26295^1b^ | amino-acid transp, put (YEU9 yeast) | ECU05_0580 |  |  |
|  | amino-acid transp, put (YEU9 yeast) | ECU05_0160 |  |  |
| 21813^1b^, 26295^1b^ | amino-acid transp, put (YEU9 yeast) | ECU09_1190 |  |  |
|  | methionine permease (MUP1 yeast) | ECU06_1480 |  |  |
|  | oligopeptide transp, PTR2 fam (OPT1 DROME) | ECU11_1050 |  |  |
|  |  |  |  |  |
|  | ***Lipid transporters*** |  | **2** | **2** |
| 22760 | phospholipid-transporting ATPase 1A (AT1A BOVIN) | ECU06_0930 |  |  |
| 23131 | phospholipid-transporting ATPase 2A (AT2A MOUSE) | ECU09_1440 |  |  |
|  |  |  |  |  |
|  | ***Purine and pyrimidine transporters*** |  | **6** | **2-5** |
| 21953^1b^, 24423^1b^, 25389^1b^, 26694^1b^ | ADP/ATP carrier protein 1 (TLCA RICPR) | ECU10_0520 |  |  |
| 21953^1b^, 24423^1b^, 25389^1b^, 26694^1b^ | ADP/ATP carrier protein 1 (TLCA RICPR) | ECU10_0540 |  |  |
| 21953^1b^, 24423^1b^, 25389^1b^, 26694^1b^ | ADP/ATP carrier protein 1 (TLCA RICPR) | ECU08_1300 |  |  |
| 21953^1b^, 24423^1b^, 25389^1b^, 26694^1b^ | ADP/ATP carrier protein 4 (TLCD RICPR) | ECU10_0420 |  |  |
|  | nicotinic acid transp, allantoate permease fam (NAT1 yeast) | ECU08_0640 |  |  |
| 24311 | nucleoside transp (YEGT ECOLI) | ECU11_1880 |  |  |
|  |  |  |  |  |
|  | ***ABC transporters*** |  | **13** | **6-7** |
| 23543 | ABC transp, EF-3 subfam (GC20 yeast) | ECU05_1190 |  |  |
|  | ABC transp, folate transp (FOL1 CAEEL) | ECU11_1600 |  |  |
| 27105 | ABC transp, MDR subfam (ABC7 MOUSE) | ECU04_0480 |  |  |
|  | ABC transp, MDR subfam (ABC7 HUMAN)^2^ | ECU01_0200 |  |  |
|  | ABC transp, MDR subfam (ABC7 HUMAN)^2^ | ECU01_1410 |  |  |
| 22828, 22496 | ABC transp, MDR subfam, mito (ATM1 yeast) | ECU11_1200 |  |  |
| 25274, 26170, 27106 | ABC transp, MDR subfam, put (Y288 THEMA) | ECU03_0240 |  |  |
| 22908 | ABC transp, MDR subfam, put (YETI SCHPO) | ECU10_1230 |  |  |
| 22678^1b^ | ABC transp, PDR5 subfam (PDR5 yeast) | ECU03_0390 |  |  |
| 22678^1b^ | ABC transp, white prt (CRD2 CANAL) | ECU08_0110 |  |  |
| 22678^1b^ | ABC transp, white prt (CDR2 CANAL) | ECU10_1520 |  |  |
| 22678^1b^, 22770 | ABC transp, white prt (WHIT DROME) | ECU07_0560 |  |  |
| 22678^1b^ | similarity to yeast ABC transporter-like fragment (PDRF yeast) | ECU11_1340 |  |  |
|  |  |  |  |  |
|  | ***Other*** |  | **3** | **2** |
| 27080, 27085 | aquaporin-like prt, MIP fam (AQPA RANES) | ECU07_0740 |  |  |
|  | frataxine-like prt (FRDA ARATH) | ECU01_1310 |  |  |
| 21982 | soma ferritin (FRIS LYMST) | ECU06_1380 |  |  |
|  |  |  |  |  |
|  | **VIII. INTRACELLULAR TRANSPORT** |  | **72** | **60** |
|  |  |  |  |  |
|  | ***Nuclear transporter*** |  | **10** | **9** |
| 26163 | ATP-dep RNA helicase involved in mRNA export (DBP5 yeast) | ECU02_1520 |  |  |
| 22825 | exportin (CRM1 SCHPO) | ECU11_0240 |  |  |
| 22379 | importin (karyopherin), ?1 sub (IMA1 SCHPO) | ECU11_1760 |  |  |
| 22739 | importin (karyopherin), β1 sub (IMB1 yeast) | ECU10_1240 |  |  |
| 26444 | nuclear GTP-Bprt (GSP1 yeast) | ECU04_1560 |  |  |
| 27452 | nucleoporin NUP155 (N155 rat) | ECU06_0470 |  |  |
| 24324 | nucleoporin NUP116 (N116 yeast) | ECU11_1820 |  |  |
|  | poly (A)+ RNA export protein (RAE1 SCHPO) | ECU08_0690 |  |  |
| 25394 | ran-specific GTPase-activating prt (RANG yeast) | ECU02_0140 |  |  |
| 24929 | ran-specific GTPase-activating prt (RANG yeast) | ECU05_0620 |  |  |
|  |  |  |  |  |
|  | ***Vesicular transport*** |  |  |  |
|  |  |  |  |  |
|  | ***ADP ribosylation factor (ARF)*** |  | **4** | **2** |
|  | GTP-Bprt, ARF fam (ARF1 yeast) | ECU10_0090 |  |  |
|  | GTP-Bprt, ARF fam (ARFM CAEEL) | ECU03_0470 |  |  |
| 24434 | GTP-Bprt, ARF fam (ARL1 DROME) | ECU08_1930 |  |  |
| 25398 | GTP-Bprt, SAR-type, ARF fam (SAR1 TRIRI) | ECU05_0090 |  |  |
|  |  |  |  |  |
|  | ***COP I coat*** |  | **6** | **6** |
| 27476 | coatomer complex, alpha sub (COPA BOVIN) | ECU01_1290 |  |  |
| 26464 | coatomer complex, beta sub (COPB yeast) | ECU08_1100 |  |  |
| 25283 | coatomer complex, beta prime sub (COPP HUMAN) | ECU08_1250 |  |  |
| 22909 | coatomer complex, gamma sub (COPG SCHPO) | ECU03_0270 |  |  |
| 23928, 24692, 26035 | coatomer complex, delta sub (COPD CAEEL) | ECU08_0340 |  |  |
| 22712 | coatomer complex, zeta sub (COPZ yeast) | ECU08_0680 |  |  |
|  |  |  |  |  |
|  | ***COP II coat*** |  | **3** | **3** |
| 22550 | COPII, protein transport prt SEC13 (SC13 PICPA) | ECU11_1450 |  |  |
| 22920 | COPII, protein transport prt SEC23 (SC23 yeast) | ECU11_0790 |  |  |
| 25850 | COPII, protein transport prt similar to SEC31 (WEB1 yeast) | ECU08_0210 |  |  |
|  |  |  |  |  |
|  | ***RAB proteins*** |  | **5** | **5** |
| 26374 | RAS-rel GTP-Bprt YPT1 (YPT1 SCHPO) | ECU03_1430 |  |  |
| 23245 | RAS-rel GTP-Bprt RAB1 (RB1B ARATH) | ECU04_0680 |  |  |
| 21682, 22510, 22709, 24062, 25976 | RAS-rel GTP-Bprt RAB5 (RAB5 TOBAC) | ECU08_0730 |  |  |
| 24669 | RAS-rel GTP-Bprt RAB6 (YPT6 yeast) | ECU09_0170 |  |  |
| 21819 | RAS-rel GTP-Bprt RAB10 (YPT2 SCHPO) | ECU09_1450 |  |  |
|  |  |  |  |  |
|  | ***Other*** |  | **17** | **14** |
| 27077 | endosomal prt of late intermediate endocytic compartment (TM21 MESAU) | ECU02_0960 |  |  |
| 24884 | GTP/GDP exchange factor for ARF (GEA1 yeast) | ECU10_1140 |  |  |
| 25982 | retrieval of ER membrane proteins from the early Golgi compartment (RER1 yeast) | ECU08_0700 |  |  |
| 22686 | synaptobrevin-rel prt (SNC2 yeast) | ECU05_0495 |  |  |
| 22572 | synaptobrevin-rel prt (SYBR ARATH) | ECU11_0980 |  |  |
| 27456 | syntaxin (STX5 HUMAN) | ECU05_0820 |  |  |
| 25379 | syntaxin-binding prt (STB2 HUMAN) | ECU02_0430 |  |  |
| 27701 | syntaxin-like prt (SFT2 yeast) | ECU09_0940 |  |  |
| 26148, 26149 | syntaxin-rel prt, put (YJD7 yeast) | ECU11_1560 |  |  |
| 24205 | transitional ER ATPase (TERA MOUSE) | ECU05_1140 |  |  |
|  | transitional ER ATPase (TERA RAT) | ECU01_1050 |  |  |
|  | vesicle-associated membrane prt (VAMP MOUSE) | ECU11_0640 |  |  |
| 24336, 24643, 26559 | vesicular integral prt (VP36 CANFA) | ECU09_0550 |  |  |
| 21730 | vesicular transport prt (P115 RAT) | ECU08_0170 |  |  |
| 22729 | vesicular transport prt, from the ER to Golgi (BOS1 yeast) | ECU07_1620 |  |  |
|  | vesicular transport prt, from the ER to Golgi (BOS1 yeast) | ECU07_0430 |  |  |
| 26410 | vesicular transport prt, Golgi retrograde traffic (VTI1 yeast) | ECU04_080 |  |  |
|  |  |  |  |  |
|  | ***Vacuolar transport*** |  |  |  |
|  |  |  |  |  |
|  | ***Vacuolar H+-ATPase (V0V1)*** |  | **11** | **8** |
| 26472 | H^+^-ATPase V_0_, 95K sub (VPH1 yeast) | ECU09_1790 |  |  |
| 25841 | H^+^-ATPase V_0_, 16K sub (VATL HUMAN) | ECU06_0190 |  |  |
|  | H^+^-ATPase V_0_, 67K sub (VATL HUMAN) | ECU07_1350 |  |  |
| 22869 | H^+^-ATPase V_1_, A sub (VATA SCHPO) | ECU11_1280 |  |  |
| 22817, 23410 | H^+^-ATPase V_1_, B sub (VATB CANTR) | ECU10_1040 |  |  |
|  | H^+^-ATPase V_1_, C sub (VATC yeast) | ECU03_0500 |  |  |
| 27254 | H^+^-ATPase V_1_, D sub (VATD SCHPO) | ECU10_0140 |  |  |
| 22660, 25371, 25709, 26264, 26718, 26763, 26825, 27631 | H^+^-ATPase V_1_, E sub (VATE NEUCR) | ECU05_0405 |  |  |
| 25392 | H^+^-ATPase V_1_, F sub (VATF RAT) | ECU03_0305 |  |  |
|  | H^+^-ATPase V_1_, 54K sub (VM13 yeast) | ECU08_0250 |  |  |
| 21958 | H^+^-ATPase V_1_, 41K sub (VATX yeast) | ECU08_1520 |  |  |
|  |  |  |  |  |
|  | ***Other*** |  | **3** | **3** |
| 22876 | vacuolar protein sorting-associated prt (VPS13 yeast) | ECU04_0700 |  |  |
| 21784 | vacuolar protein sorting-associated prt (VP45 yeast) | ECU06_1460 |  |  |
| 21703 | vacuolar protein sorting-associated prt, put (YAB3 SCHPO) | ECU03_0900 |  |  |
|  |  |  |  |  |
|  | ***Extracellular transport (secretion)*** |  | **3** | **3** |
| 22848 | RAB-GDP dissociation inhibitor (GDI1 yeast) | ECU11_0350 |  |  |
| 21968 | RAB-GDP dissociation inhibitor, put (YD4C SCHPO) | ECU02_1410 |  |  |
| 25423 | recessive suppressor of secretory defect (RSD1 yeast) | ECU03_1160 |  |  |
|  |  |  |  |  |
|  | ***Cellular import*** |  | **6** | **4** |
| 26021 | β1-adaptin, large sub (ADB6 yeast) | ECU06_0770 |  |  |
| 24208 | adaptin, large sub (AP47 MOUSE) | ECU02_0540 |  |  |
| 22341 | adaptin, small sub (AP19 yeast) | ECU08_0150 |  |  |
|  | dynamin-like vacuolar protein sorting prt (VPS1 yeast) | ECU10_1700 |  |  |
|  | dynamin-rel prt (DYN3 RAT) | ECU01_1210 |  |  |
| 24603 | vesicular fusion prt SEC18 (SC18 yeast) | ECU03_1410 |  |  |
|  |  |  |  |  |
|  | ***Other intracellular-transport activities*** |  | **4** | **3** |
| 24199, 27468 | DNAJ homolog 2 involved in mitochondrial protein import (MAS5 yeast) | ECU07_0760 |  |  |
| 22338 | similarity to 14.3.3 prt (143B RAT) | ECU03_1010 |  |  |
| 27694 | translocase involved in protein import, mito (IM22 SCHPO) | ECU07_0240 |  |  |
|  | translocase involved in protein import, mito (OM70 yeast) | ECU09_0870 |  |  |
|  |  |  |  |  |
|  | **IX CELLULAR ORGANIZATION AND BIOGENESIS** |  | **69** | **42** |
|  |  |  |  |  |
|  | ***Cell surface*** |  | **7** | **1** |
|  | glypican, w. sim (GPC4 MOUSE) | ECU01_0110 |  |  |
|  | glypican, w. sim (GPC4 MOUSE) | ECU01_1500 |  |  |
|  | similarity to HDL-Bprt (HBP HUMAN) | ECU06_1470 |  |  |
|  | similarity to mammalian integral membrane prt (polyposis locus prt 1) (DP1 MOUSE) | ECU09_1950 |  |  |
| 22556 | similarity to Plasmodium membrane prt (A412 PLAFA) | ECU11_1320 |  |  |
|  | similarity to Saccharomyces integral membrane prt (ORM1 yeast) | ECU11_1150 |  |  |
|  | spore wall protein SWP1 (SWP1 ENCCU) | ECU10_1660 |  |  |
|  | ***Cytoskeleton*** |  |  |  |
|  |  |  |  |  |
|  | ***Actin cytoskeleton*** |  | **12** | **11** |
| 24676 | α-actinin, non muscle (AACN CHICK) | ECU09_0290 |  |  |
| 24869 | actin (ACT1 STRFN) | ECU01_0460 |  |  |
|  | actin-like 53K prt (ACT BRUMA) | ECU04_1090 |  |  |
| 22814 | actin-rel prt (ARP5 yeast) | ECU05_0400 |  |  |
| 27244 | actin depolymerizing factor (ADF BRANA) | ECU10_0360 |  |  |
| 24862, 25422 | calponin H2-like prt (MP20 DROME) | ECU03_1150 |  |  |
| 23707 | coil-coiled myosin-like prt, put (YAV4 SCHPO) | ECU09_1690 |  |  |
| 24662 | leucine-rich repeat prt, villin/gelsolin fam (FLII DROME) | ECU09_0200 |  |  |
| 27251 | LIM domain-containing prt (SLI3 MOUSE) | ECU11_0340 |  |  |
| 22793 | myosin heavy chain, non-muscle type A (MYST RABI) | ECU04_1000 |  |  |
| 21731 | myosin heavy chain, non-muscle isoform 2 (MYS2 yeast) | ECU09_1970 |  |  |
| 24348 | myosin reg light chain (MLRH CAEEL) | ECU03_1570 |  |  |
|  |  |  |  |  |
|  | ***Microtubule cytoskeleton*** |  | **15** | **7-8** |
| 21799 | α-tubulin (TBA HAECO) | ECU07_1190 |  |  |
|  | α-tubulin folding cofactor D (ALF1 yeast) | ECU06_0220 |  |  |
| 21737, 21799, 22227, 22318, 22349, 23088, 23457, 23644, 23673, 23956, 24030, 24037, 24107, 24125, 24403, 24623, 24804, 26273, 26976, 27014, 27391 | β-tubulin (TBB4 XENLA) | ECU03_0820 |  |  |
| 22249, 26023 | γ-tubulin (TBG XENLA) | ECU08_0670 |  |  |
| 25963 | binds microtubules (BIM1 yeast) | ECU01_1030 |  |  |
|  | caltractin (centrin) (CATR CHLRE) | ECU07_1230 |  |  |
|  | dynein heavy chain (DYHC yeast) | ECU10_0640 |  |  |
| 22501 | dynein light chain 1 (DYL1 yeast) | ECU01_0260 |  |  |
| 25273^1b^, 27248^1b^ | kinesin-rel prt (KIP2 yeast) | ECU10_1000 |  |  |
| 25273^1b^, 27248^1b^ | kinesin-rel prt (KINH STRPV) | ECU08_0890 |  |  |
| 25273^1b^, 27248^1b^ | kinesin-rel prt (mitotic) (KIF2 HUMAN) | ECU11_0470 |  |  |
| 25273^1b^, 27248^1b^ | kinesin-rel prt (organelle transport) (KIF4 MOUSE) | ECU05_0060 |  |  |
| 25273^1b^, 27248^1b^ | kinesin-like DNA-Bprt (KID HUMAN) | ECU11_2020 |  |  |
| 25273^1b^, 27248^1b^ | kinesin-like prt A (carboxy-terminal) (KATA ARATH) | ECU10_0320 |  |  |
| 22580, 22587, 22876, 23222, 25276 | ser/thr protein phosphatase involved in microtubule organization (PP4 HUMAN) | ECU10_1280 |  |  |
|  |  |  |  |  |
|  | ***Endoplasmic reticulum and Golgi*** |  | **5** | **4** |
| 24138, 24320 | ER membrane prt degrading misfolded ER luminal proteins (DER1 yeast) | ECU11_1920 |  |  |
| 26853 | ER membrane prt regulating the retention of ER resident proteins (G25L CANFA) | ECU03_1260 |  |  |
| 27692 | Golgi GDP-mannose transp (GOG5 yeast) | ECU03_0150 |  |  |
|  | Golgi membrane prt (YAT2 SCHPO) | ECU01_0530 |  |  |
| 26453 | sorting nexin (Golgi retention) (SNX3 yeast) | ECU05_0840 |  |  |
|  |  |  |  |  |
|  | ***Nucleus*** |  | **6** | **3** |
|  | nuclear envelope prt, w.sim (NDC1 yeast) | ECU02_0090 |  |  |
|  | nuclear movement prt (NUDC EMENI) | ECU01_1280 |  |  |
| 27437 | nuclear prt (RG1 yeast) | ECU05_0710 |  |  |
| 25570 | nuclear prt, NHP2/RS6 family (NHPX yeast) | ECU07_0460 |  |  |
|  | nuclear prt, put (YKJ5 yeast) | ECU08_0570 |  |  |
| 23125 | nuclear scaffold prt (BIMA EMENI) | ECU11_1160 |  |  |
|  |  |  |  |  |
|  | ***Chromosome structure*** |  | **18** | **14** |
| 22325 | ATPase component of the two-subunit chromatin remodeling factor (ISWI DROME) | ECU10_1320 |  |  |
|  | chromatin assembly factor 1, 60K sub (CAC2 yeast) | ECU08_1260 |  |  |
| 22765, 26538 | chromatin structure modulator (SPT6 yeast) | ECU07_1690 |  |  |
| 26443 | chromosome condensation regulator (RCC SCHPO) | ECU04_1500 |  |  |
| 25578 | condensin sub (CND1 SCHPO) | ECU02_1240 |  |  |
| 25984 | condensin sub (CND3 SCHPO) | ECU08_0610 |  |  |
| 25828 | heterochromatin prt (CBX1 HUMAN) | ECU03_0180 |  |  |
|  | histone-Bprt N1/N2 (HIBN XENLA) | ECU05_0370 |  |  |
| 27207 | histone H2A (H2A1 TETPY) | ECU02_0720 |  |  |
| 26025 | histone H2B(H2B ENTIN) | ECU08_0410 |  |  |
| 27200^1b^ | histone H3 (H3 CAEEL) | ECU03_1460 |  |  |
| 27200^1b^ | histone H3 (H3 VOLCA) | ECU09_0450 |  |  |
| 27695 | histone H4 (H4 DROME) | ECU09_0440 |  |  |
| 25426, 25702, 25752, 27337, 27363, 27667 | HMG1 (HMG1 CHICK) | ECU06_1270 |  |  |
| 25419, 25570 | HMG2 (NHP2 yeast) | ECU11_0940 |  |  |
|  | similarity to zuotin (ZUO1 yeast) | ECU01_0370 |  |  |
| 22194, 22424, 22639, 22938, 22964, 23521, 23751, 23808, 23815, 24148, 24414, 24559, 24621, 24708, 24900, 24954, 25225, 25587, 26219, 26884, 27367 | structure-specific recognition protein (SSRP MOUSE) | ECU07_1010 |  |  |
| 24323 | histone-H1 | ECU11_1790 |  |  |
|  |  |  |  |  |
|  | ***Other*** |  | **6** | **2** |
|  | belongs to the cytochrome b5 fam, put (YDAA SCHPO) | ECU01_1115 |  |  |
|  | belongs to the ERV1/ARL family, mito (ERV1 yeast) | ECU06_1090 |  |  |
|  | polar tube protein 1 (PTP1 ENCCU) | ECU06_0250 |  |  |
| 26400 | polar tube protein 2 (PTP2 ENCCU) | ECU06_0240 |  |  |
|  | similarity to *E. coli* periplasmic divalent cation tolerance prt (CUTA ECOLI) | ECU04_1360 |  |  |
| 26075, 26413 | similarity to chloroplast 20Kda- RNP (R028 SPIOL) | ECU01_0840 |  |  |
|  |  |  |  |  |
|  | **X. CELLULAR COMMUNICATION / SIGNAL TRANSDUCTION** |  | **26** | **17** |
|  |  |  |  |  |
|  | ***Kinases*** |  | **12** | **8** |
| 24351 | calmodulin dep protein kinase (BYR2 SCHPO) | ECU03_0630 |  |  |
| 21713, 22789, 24213, 25561, 27474 | cAMP-dep ser/thr protein kinase, α sub (PKX1 HUMAN) | ECU07_0520 |  |  |
|  | cAMP-dependent protein kinase, ? sub (KAPR DROME) | ECU08_0930 |  |  |
| 25148^1b^ | phosphatidylinositol-3-kinase, carboxy-terminal (PI3K ARATH) | ECU10_0590 |  |  |
| 25148^1b^ | phosphatidylinositol 4-kinase, cat sub (PIK1 yeast) | ECU03_1100 |  |  |
| 25148^1b^ | phosphatidylinositol 4-kinase, alpha sub, carboxy-terminal (STT4 yeast) | ECU11_0450 |  |  |
| 26450 | phosphatidylinositol-4-phosphate 5-kinase (MSS4 yeast) | ECU03_0330 |  |  |
| 25523 | protein kinase, PI3/PI4 fam (ERS1 yeast) | ECU02_1130 |  |  |
| 23269 | protein kinase, PI3/PI4 fam, put (YAMB SCHPO) | ECU05_0540 |  |  |
| 27474 | protein kinase C (KPC2 ALPCA ) | ECU01_1320 |  |  |
|  | ser/thr protein kinase, MNB/DYRK subfam (YAK1 yeast) | ECU11_0180 |  |  |
| 21713 | SNF1-related protein kinase (KI10 ARATH) | ECU08_1480 |  |  |
|  |  |  |  |  |
|  | ***Guanine nucleotide-binding proteins*** |  | **7** | **6-7** |
| 25958 | β-transducin repeat-containing prt (TRCB XENLA) | ECU05_1010 |  |  |
| 25381, 27245^1b^ | GTP-Bprt, RHO subfam (RHO1 CANAL) | ECU02_0410 |  |  |
| 27245^1b^ | GTP-Bprt, RHO subfam (RHO1 DROME) | ECU10_0350 |  |  |
| 25513, 26038, 26308, 26929, 27222 | guanine nucleotide-Bprt, β sub (GBB2 MOUSE) | ECU05_0740 |  |  |
| 26041 | guanine nucleotide-Bprt, β sub (GBLP DROME) | ECU08_0310 |  |  |
| 23274 | guanine nucleotide-Bprt, β sub (GBLP SCHPO) | ECU09_1770 |  |  |
| 26463 | guanine nucleotide-Bprt, β sub (GBLP TOBAC) | ECU08_1110 |  |  |
|  |  |  |  |  |
|  | ***Other*** |  | **7** | **3** |
| 22582 | ankyrin repeat-containing prt (ANK1 MOUSE) | ECU03_1590 |  |  |
|  | ankyrin repeat-containing prt (YA2A SCHPO) | ECU10_0720 |  |  |
|  | calmodulin (CALM NEUCR) | ECU09_1220 |  |  |
| 27436 | calmodulin-binding protein, w.sim (CN1A BOVIN) | ECU05_0700 |  |  |
| 24349 | phosphatidylinositol transfer prt (PPI2 MOUSE) | ECU05_0930 |  |  |
|  | similarity to adenylate cyclases of class 3, put (Y891 MYCTU) | ECU06_0960 |  |  |
|  | translocates to the nucleus after nutrient stimulation (ZPR1 HUMAN) | ECU04_1480 |  |  |
|  |  |  |  |  |
|  | **XI. CELL RESCUE, DEFENSE, CELL DEATH AND AGING** |  | **22** | **16** |
|  |  |  |  |  |
|  | ***DNA repair (direct repair, base excision...)*** |  | **9** | **6** |
|  | 3-methyl-adenine DNA glycosylase (3MG HUMAN) | ECU05_1590 |  |  |
|  | 3-methyl-adenine DNA glycosylase (3MGH BACSU) | ECU11_0140 |  |  |
| 23306, 24147, 25981 | 8-oxoguanine DNA glycosylase (OGG1 yeast) | ECU08_0770 |  |  |
|  | DNA excision repair prt (ERC1 MOUSE) | ECU10_1330 |  |  |
| 22037, 22238, 22661, 23935, 23961, 25584, 25789, 26265, 26345, 26554, 26719, 26899, 27023, | DNA lyase of class II (APE1 HUMAN) | ECU06_1360 |  |  |
| 27527 | DNA lyase, endonuclease 3 (END3 SCHPO) | ECU08_0880 |  |  |
| 22824 | DNA lyase, endonuclease 4 (APN1 CAEEL) | ECU11_1550 |  |  |
| 22583 | DNA repair protein RAD51 RA51 CHICK) | ECU11_0820 |  |  |
| 21734 | structure-specific nuclease (RAD2 SCHPO) | ECU03_1080 |  |  |
|  |  |  |  |  |
|  | ***Detoxification*** |  | **10** | **8** |
|  | adrenodoxin (ferredoxin) (FER2 RICPR) | ECU07_0600 |  |  |
| 25378 | glutathione peroxidase (GSHH RAT) | ECU02_0440 |  |  |
| 27240 | hydroxyacyl glutathione hydrolase (GLO2 yeast) | ECU02_0580 |  |  |
| 22568 | manganese superoxide dismutase, mito (SODM BACCA) | ECU11_1080 |  |  |
| 26091 | NADPH-cytochrome P450 RDase (NCPR RABIT) | ECU05_0240 |  |  |
| 22870 | NADPH-adrenodoxin oxidoRDase (ADRO yeast) | ECU11_1310 |  |  |
| 27697 | thioredoxin, H-type (THIH ORYZA) | ECU01_0930 |  |  |
| 26089 | thioredoxin, put (YPD3 CAEEL) | ECU01_0720 |  |  |
|  | thioredoxin peroxidase (TDX FASHE) | ECU03_1190 |  |  |
| 26258 | thioredoxin RDase (TRXB BORBU) | ECU01_0680 |  |  |
|  |  |  |  |  |
|  | ***Other*** |  | **3** | **2** |
| 25407 | DNA damage-responsive protein kinase (ALK1 yeast) | ECU03_0890 |  |  |
|  | longevity assurance prt 1 (LAG1 SCHPO) | ECU08_1350 |  |  |
| 22875 | similarity to human cell apoptosis-related gene TFAR19 (TF19 HUMAN) | ECU11_1360 |  |  |

^1a^ *E. bieneusi* protein is a homolog of more than 1 *E. cuniculi* protein that is multi-copy; counted as same

number of *E. cuniculi* proteins.

^1b^ *E. bieneusi* protein is a homolog of more than 1 *E. cuniculi* protein, but is only counted as 1.

^2^ *E. cuniculi* multi-copy gene.
