# Supplementary material for: Genomic Survey of the Non-Cultivatable Opportunistic Human Pathogen, Enterocytozoon bieneusi
Source: PLoS Pathog. 2009 Jan 9;5(1):e1000261. doi: 10.1371/journal.ppat.1000261 (PMC2607024; doi:10.1371/journal.ppat.1000261)
Supplement: Table S5 — Summary of the E. bieneusi contig data. (0.03 MB DOC) [file ppat.1000261.s007.doc]

Table S5. Summary of the *E. bieneusi* contig data.

| Length of contigs (bases) | Number of contigs | Total bases represented^1^ | %AT |
| --- | --- | --- | --- |
| >100 kb | 4 | 476,191 | 74.3 |
| >50 kb | 11 | 1,021028 (544,837) | 74.0 |
| >20 kb | 27 | 1,479,459 (458,431) | 76.1 |
| >10 kb | 36 | 1,615,303 (135,844) | 76.9 |
| >5 kb | 46 | 1,688,003 (72,700) | 74.6 |
| >4 kb | 49 | 1,701,767 (13,764) | 74.3 |
| >2 kb | 136 | 1,912,006 (210,239) | 62.0 |
| < 2 kb | 1606 | 1,947,646 | 58.7 |

^1^ Total bases represented in all contigs greater than the length indicated for the 2-100 kb groups and total bases for contigs < 2 kb. In parentheses, number of bases for that size range (i.e. in the >50 kb group, 544,837 bases are represented by contigs with lengths greater than 50 kb but less than 100 kb).
